# Optoelectronic Properties of Triply Twisted Möbius Carbon Nanobelt and the Design of Its Isomeric Nanomaterials

**DOI:** 10.3390/molecules29194621

**Published:** 2024-09-29

**Authors:** Cailian Yao, Tao Wang

**Affiliations:** College of Science, Liaoning Petrochemical University, Fushun 113001, China

**Keywords:** triply twisted Möbius carbon nanobelt, isomeric nanomaterials, OPA, TPA, ECD

## Abstract

The triply twisted Möbius carbon nanobelt (TMCNB) possesses an extremely distinctive geometric and electronic structure and therefore is anticipated to demonstrate outstanding optical properties. In this paper, through integrating quantum chemical calculations and wave function analysis approaches, in-depth studies are conducted on the one-photon absorption (OPA) and two-photon absorption (TPA) characteristics, aromaticity, and circular dichroism of the TMCNB. Inspired by this structure, we further deform it to construct a novel structure, MCNB2, and verify the stability of this structure, thereby confirming its rationality. Since variations in structure will result in dissimilar optical properties, we also undertake theoretical analyses on the absorption properties and circular dichroism of MCNB2. The outcomes of this study offer a significant theoretical foundation for the design and construction of chiral optoelectronic materials.

## 1. Introduction

Topological molecular carbons, such as twisted and knotted carbon nanostructures, have attracted significant attention due to their aesthetic appeal and potential applications in fields like electronics, photonics, and spintronics. Möbius carbon nanobelts (MCNBs), as a type of these, have unique topological chirality and potential application values. However, the precise synthesis of such molecules, such as MCNBs with specific twist numbers, is a major challenge in synthetic organic chemistry, requiring precise control of twisting and strain.

The Möbius band was independently discovered by the 19th century German mathematicians August Ferdinand Möbius and Johann Benedict Listing as a topological structure. It has only one surface and one edge. If it is cut along the centerline of the band, the resulting structure is still a longer Möbius band. This structure has a profound influence in mathematics and physics and has inspired scientists to explore similar nanostructural scales [1].

At the end of the 20th century and the beginning of the 21st century, with the advancement of computer simulation technologies, scientists theoretically foresaw the possibility of twisting carbon nanobelts into Möbius bands. These early theoretical studies, through the methods of quantum mechanics and computational chemistry, predicted the structure and stability of Möbius carbon nanobelts [2,3,4].

In 2003, the German scientist Herges successfully synthesized a stable “Möbius” aromatic compound, which not only verified the existence of Möbius aromaticity but also broadened scientists’ understanding of aromatic compounds and topological chemistry [5]. In 2014, Gaston R. Schaller and colleagues proposed a topological strategy of converting “twist” into “writhe” to synthesize multiply twisted cyclic π-systems. Based on this strategy, they successfully synthesized a triply twisted dehydroannulene [6]. The Möbius carbon nanobelt was first successfully synthesized by Tanaka from the University of Tokyo in 2017, representing a significant breakthrough in the field of carbon nanomaterials. It exhibits a closed structure with a single side and a single surface, opening up a new direction for the research of carbon nanobelts [7]. In 2018, Guillaume Naulet synthesized cyclic tris-[5]helicenes through an efficient synthetic strategy based on the Perkin reaction for assembling building blocks into large conjugated macrocycles and the photocyclization reaction for rigidifying these flexible precursors, as presented in this article [8]. In 2022, Wei Fan et al. for the first time rapidly synthesized the first fully sp^2^-hybridized, triply twisted Möbius carbon nanobelt through a synthetic route of Suzuki coupling-mediated macrocyclization, Bi(OTf)_3_-catalyzed ethylene ether cyclization, and oxidative dehydrogenation. It was found to have obvious local aromatic characteristics and showed chiral optical properties [9].

OPA and TPA are two important optical processes that have extensive applications in optical materials and nonlinear optical applications. Single-photon absorption is widely used in conventional optical devices [10,11,12], while two-photon absorption, due to its nonlinear characteristics, has unique advantages in high-resolution imaging and high-density data storage [13,14,15,16]. Chiral molecules refer to those molecules that cannot overlap with their mirror images. This asymmetry gives them chirality. The chiral center and its formation mechanism in molecular systems are of great significance in multiple fields, especially in chemistry, pharmacy, biology, and material science [17,18,19,20]. Local aromaticity refers to the characteristic in which some structures or regions in a molecule exhibit aromaticity, while the entire molecule may not be fully aromatic. In some molecules, due to the particularity of the structure, there may exist local electron delocalization and cyclic conjugated systems, giving these local regions a relatively stable electronic structure and aromaticity characteristics. This local aromaticity can affect the chemical properties, reaction activity, and physical properties of the molecule, etc. [21,22,23].

In this work, we studied the OPA and TPA properties, as well as the properties of chirality, anisotropy of the induced current density (AICD) [24], the iso-chemical shielding surface (ICSS) [25] and the localized orbital locator (LOL) [26], based on DFT [27] calculations. This is of great significance for understanding the electronic transition mechanism, chiral interaction mechanism, degree of electron delocalization, and local aromaticity. Then, by twisting the benzene ring chains in three directions, a new Möbius carbon nanobelt structure MCNB2 was obtained, and the stability of this structure as well as its linear and nonlinear optical properties were studied.

## 2. Results and Discussion

### 2.1. The Physical Mechanism of the Photoelectric Properties of TMCNB

#### 2.1.1. Structure and Properties of TMCNB

**Figure 1:**
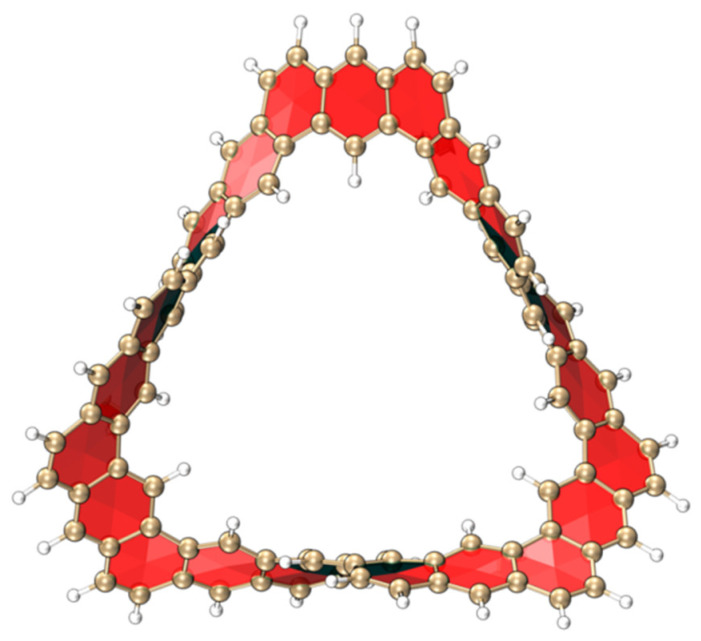
Three-dimensional structure diagram of TMCNB.

Figure 1 shows the 3D structure of the TMCNB. This structure is connected by 24 conjugated bonds and forms a unique Möbius carbon nanobelt structure through three twists. This section will focus on the linear and nonlinear optical properties of this structure as well as its chirality and aromaticity characteristics.

**Figure 2:**
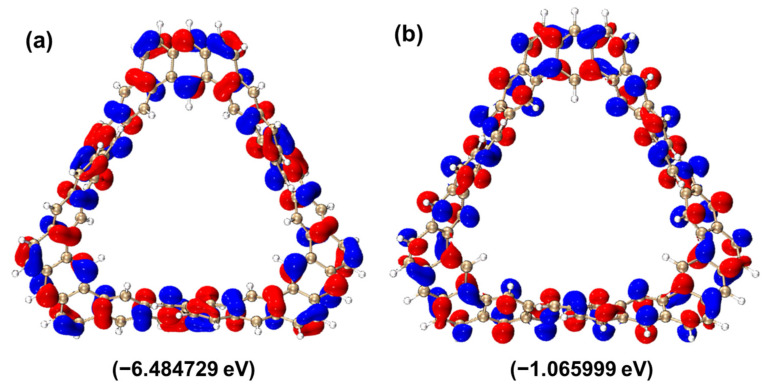
Isosurface diagram of the HOMO (**a**)and LUMO (**b**) molecular orbitals of TMCNB. The isovalue is 0.005.

The higher the energy level of the highest occupied molecular orbital (HOMO), the lower the ionization energy, which means that the molecule is more likely to lose electrons. The lower the energy level of the lowest unoccupied molecular orbital (LUMO), the easier it is for electrons to be captured by this orbital. It reflects the difficulty of electron transition from the HOMO to the LUMO. The larger this energy difference, the higher the energy barrier that electrons need to overcome for the transition, and the less likely the transition is; conversely, the smaller the energy difference, the easier the electron transition occurs. This energy difference is one of the important characteristics of the molecular electronic structure and has an important influence on the optical and electrical properties of the molecule. From Figure 2, the electrons and holes of HOMO and LUMO are represented by red and blue, respectively. The HOMO energy level of the TMCNB is −6.484729 eV, the LUMO energy level is −1.0660 eV, and the HOMO–LUMO gap is 5.4187 eV. In the isosurface diagrams of HOMO and LUMO, it can be found that the isosurfaces of electrons and holes are uniformly distributed on the conjugated plane, which reflects the characteristics of π excitation. Therefore, this excitation is a π–π* excitation with local excitation characteristics.

#### 2.1.2. OPA and TPA Spectral Analysis

**Figure 3:**
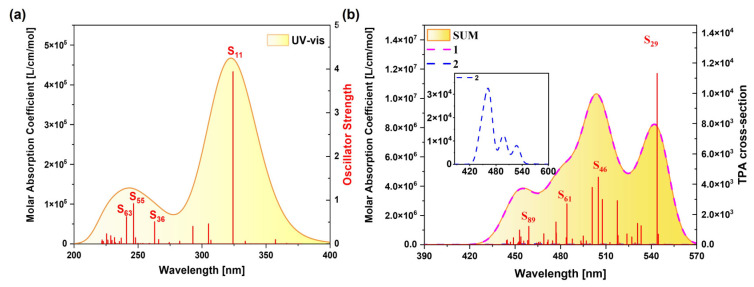
OPA spectrum (**a**) and TPA spectrum (**b**) of TMCNB.

The spectral characteristics of a molecule are closely related to its own structure. In the OPA spectrum, the molecule jumps from the ground state to the excited state after absorbing one photon, and the position and intensity of the absorption peak reflect the energy difference between different energy levels in the molecule and the transition probability. The TPA spectrum requires two photons to be absorbed simultaneously, and the position and intensity of its absorption peak are related to the second-order nonlinear optical properties of the molecule and can also provide information about the molecular structure. Therefore, we can observe the excitation characteristics of the molecule more intuitively by plotting the OPA spectrum and the TPA spectrum. Figure S1 shows the OPA spectrum of the TMCNB prepared in the experiment, and the calculated results are basically consistent with the experimental results, fully demonstrating the accuracy of the calculation method. To highlight the core TMCNB structure, we replaced the functional groups in the original structure with H to obtain the TMCNB structure and carried out a series of theoretical calculations and analyses on this structure in vacuum. Figure 3a shows theone-photon absorption spectrum of the TMCNB, and the two absorption peaks are in the range of 200–400 nm, belonging to the ultraviolet region. The absorption peak at 322 nm is the strongest, mainly contributed by the excited state S_11_, and the weaker absorption peaks are mainly contributed by the excited states S_36_, S_55_ and S_63_. Figure 3b shows the TPA spectrum, and its absorption wavelength is in the range of 420–570 nm, which is the visible light region. The TPA absorption spectrum has three absorption peaks.

**Figure 4:**
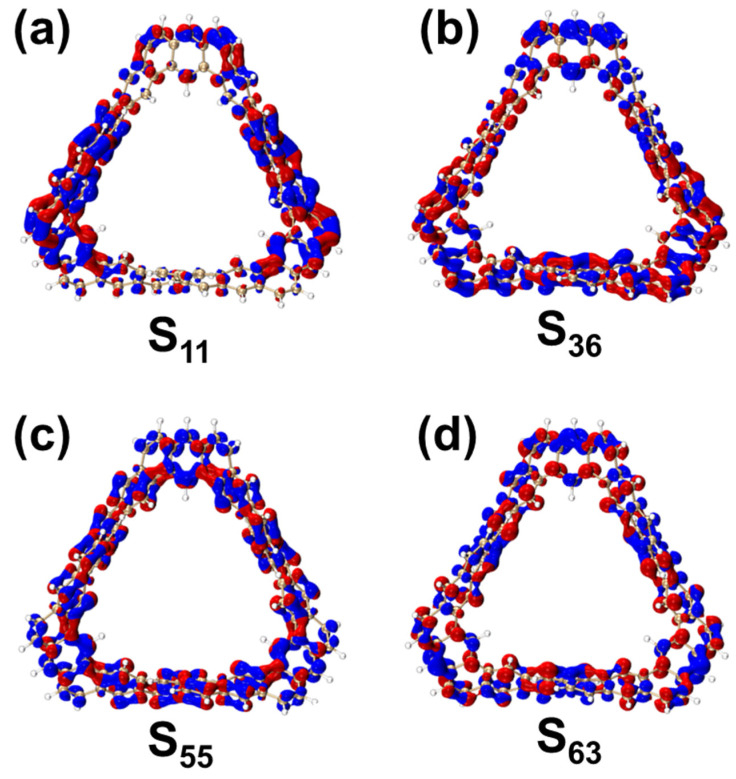
CDD diagrams of TMCNB under S_11_ (**a**), S_36_ (**b**), S_5_ (**c**) and S_63_ (**d**). In the CDD diagram, blue represents holes and red represents electrons. The isovalue is 0.005.

In the OPA spectrum, for the S_11_ state of the TMCNB, the electron and hole distributions are primarily localized on the left and right sides of the structure, and their distribution is uniform, exhibiting the characteristics of local excitation, as shown in Figure 4a. Under the other three excited states, electrons and holes are distributed across the entire structure, which also indicates local excitation. Additionally, the isosurfaces of electrons and holes are both positioned on the upper and lower sides of the benzene ring, corresponding to π^out^-π^out*^ (out-of-plane π) excitation. The TDM diagram further reveals that the transition density is concentrated along the diagonal, confirming that the nature of the excitation in these states is local excitation, as shown in Figure S2a–d.

**Figure 5:**
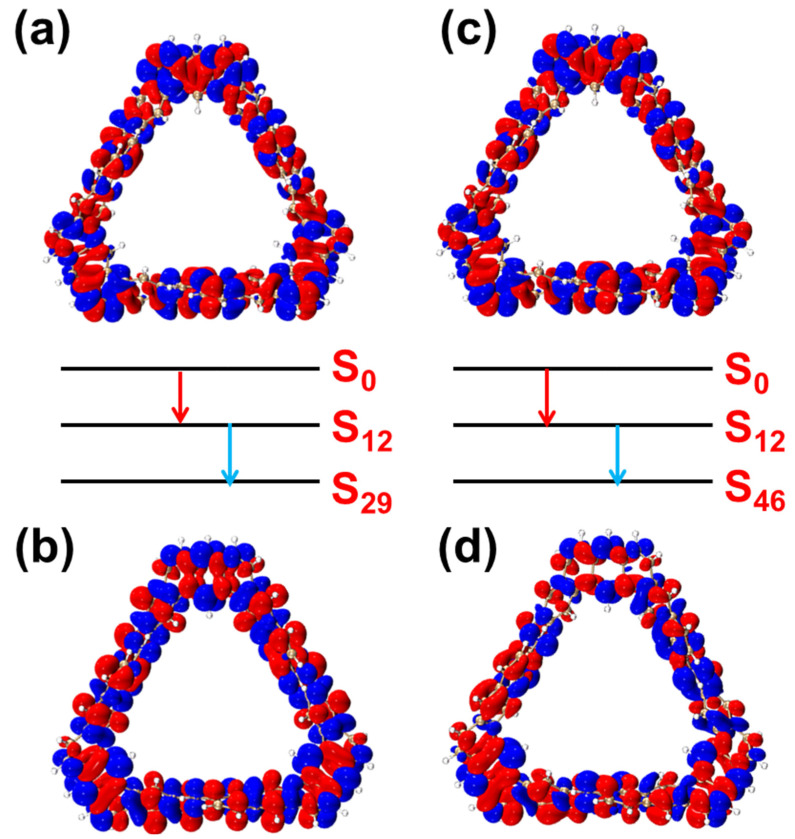
Electron–hole pair density of the two-step transition of TMCNB in S_29_, from the ground state to the intermediate state (**a**) and from the intermediate state to the final state (**b**). Electron–hole pair density of the two-step transition of TMCNB in S_46_, from the ground state to the intermediate state (**c**) and from the intermediate state to the final state (**d**) In the electron-hole pair density diagram, blue represents holes and red represents electrons. The red arrows represent the first step and the blue arrows represent the second step. The isovalue is 0.0006.

To further investigate the nonlinear optical properties of the TMCNB, we conducted an in-depth analysis of the two-photon absorption (TPA) excitation characteristics. From the TPA spectrum, it is evident that the first step of the transition dominates the overall process, while the intensity of the second-step transition is significantly weaker. Further analysis using the TDM and electron–hole pair density will provide a more detailed understanding. The contribution of electron–hole pairs in the TPA-excited state offers a clearer reflection of the charge transfer process, from the ground state to the intermediate state and ultimately to the final state, thereby highlighting both local and global excitation characteristics. Figure 5 and Figure S3 present the electron–hole pair density and TDM diagrams for the two-step transition process of the TMCNB in the excited states S_29_ and S_46_. For the first transition process of S_29_, that is, S_0_→S_12_, the bright region along the main diagonal in the TDM diagram is highly prominent, indicating that this transition is characterized by local excitation (Figure S3a). In contrast, the transition density of the second-step transition is spread across different regions, suggesting a charge transfer nature. The transition properties of *S*_46_ follow a similar pattern to those observed in *S*_29_.

**Figure 6:**
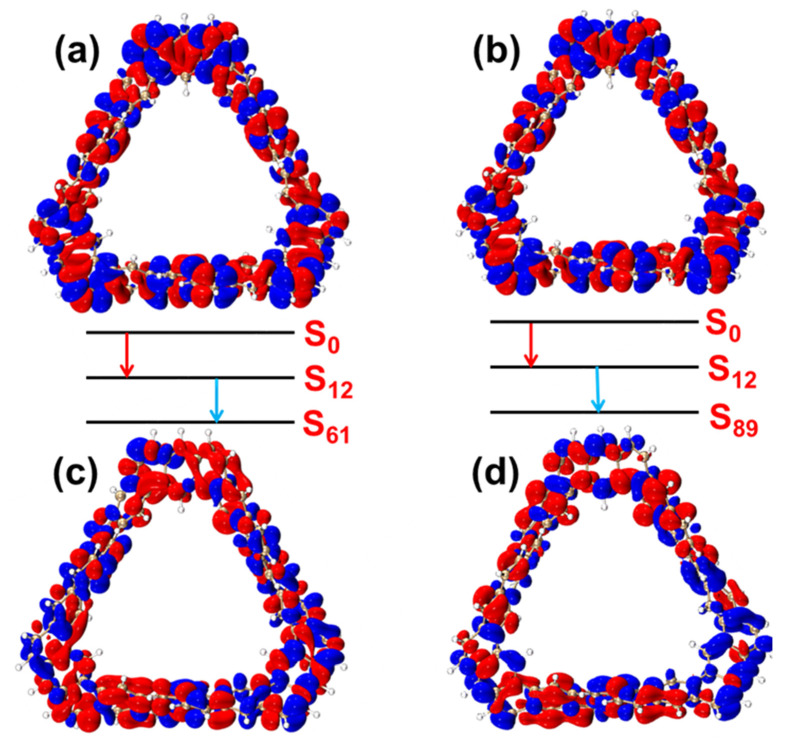
Electron–hole pair density of the two-step transition of TMCNB in S_61_, from the ground state to the intermediate state (**a**) and from the intermediate state to the final state (**b**). Electron–hole pair density of the two-step transition of TMCNB in S_89_, from the ground state to the intermediate state (**c**) and from the intermediate state to the final state (**d**). In the electron-hole pair density diagram, blue represents holes and red represents electrons. The red arrows represent the first step and the blue arrows represent the second step. The isovalue is 0.0006.

Figure 6 shows the TDM diagrams and electron–hole pair density diagrams of the two-step transition process of the TMCNB in the excited states S_61_ and S_89_. For S_61_, its first transition is similar to that of S_29_ and S_46_, exhibiting local excitation characteristics (Figure 6a). In the second step transition, the transition density is distributed across various regions. The electron–hole pair density diagram shows a higher concentration of electrons on the left side of the structure and holes on the right side, indicating charge transfer from the left to the right side of the structure. In the case of S_89_, holes are mainly distributed in the lower right corner, and electrons undergo transitions from this position to other positions, showing the characteristics of charge transfer.

#### 2.1.3. Chiral Physical Mechanism of Electronic Circular Dichroism

The mechanism of the asymmetric electromagnetic interaction between molecules and light is the source of molecular ECD. Molecular polarization can affect molecular chirality by influencing TEDM\TMDM. In this section, we explain the formation mechanism of molecular chirality by visualizing TEDM\TMDM. The intensity of ECD can be expressed as:

(1) $$I\propto \langle \varphi_{j}\left|\mu_{e}\right|\varphi_{i}\rangle \langle \varphi_{j}\left|\mu_{m}\right|\varphi_{i}\rangle B$$

where $\mu_{e}$ is the transition electric dipole moment, $\mu_{m}$ is the transition magnetic dipole moment, φ is the orbital wave function, i and j are the occupied orbital numbers and B is the magnetic induction intensity. ECD is the asymmetric response of the molecule to the electromagnetic interaction. The ECD intensity can be expressed by the tensor product of TEDM and TMDM. TEDM can be expressed as:

(2) $$D_{x}^{\mu }=P_{\mu \mu }^{tran}\langle \chi_{\mu }\left|-x\right|\chi_{\mu }\rangle +\frac{\sum_{\mu \neq \nu }\left[P_{\mu \nu }^{\mathrm{tran}}\langle \chi_{\mu }\left|-x\right|\chi_{\nu }\rangle +P_{\nu \mu }^{\mathrm{tran}}\langle \chi_{\nu }\left|-x\right|\chi_{\mu }\rangle \right]}{2}$$

TMDM can be expressed as:

(3) $$M_{x}^{\mu }=P_{\mu \mu }^{tran}\langle \chi_{\mu }\left|x\frac{d}{dy}-y\frac{d}{dx}\right|\chi_{\mu }\rangle +\frac{\sum_{\mu \neq \nu }\left[P_{\mu \nu }^{tran}\langle \chi_{\mu }\left|x\frac{d}{dy}-y\frac{d}{dx}\right|\chi_{\nu }\rangle +P_{\nu \mu }^{tran}\langle \chi_{\nu }\left|x\frac{d}{dy}-y\frac{d}{dx}\right|\chi_{\mu }\rangle \right]}{2}$$

**Figure 7:**
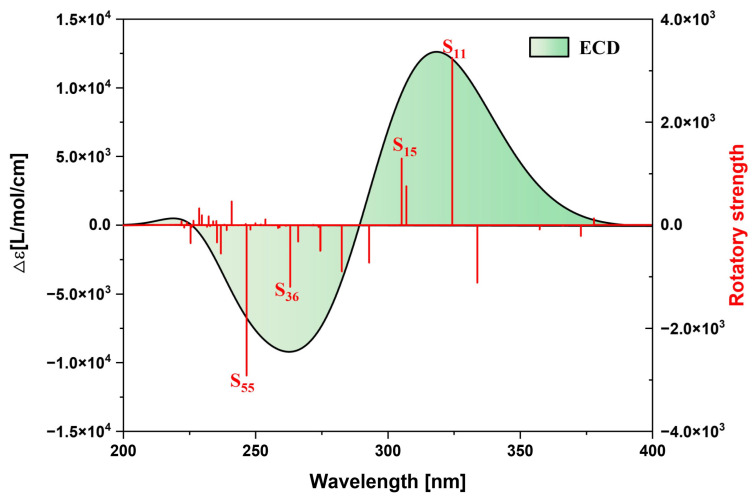
ECD spectrum of TMCNB.

**Figure 8:**
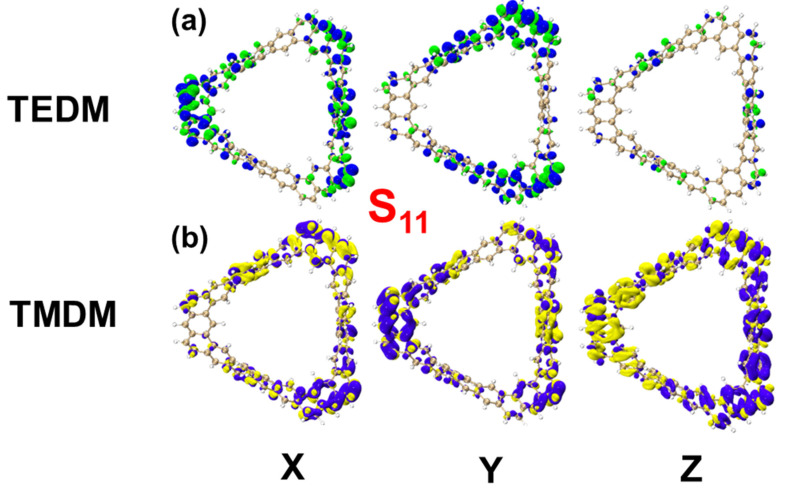
Isosurfaces of TEDM/TMDM of TMCNB in the excited states of S_11_ (**a**,**b**). The blue and green isosurfaces represent the positive and negative TEDM, respectively, and the yellow and purple isosurfaces represent the positive and negative TMDM, respectively.

Figure 7 shows the ECD spectrum of the TMCNB, displaying two positive peaks and one negative peak in the range of 200–400 nm. The primary positive peak is mainly attributed to the excited states S_11_ and S_15_, while the negative peak is mainly contributed to by the excited states S_36_ and S_55_. To further analyze the electromagnetic interaction of the TMCNB in these excited states, a visual examination of TEDM and TMDM was carried out. As shown in Figure 8, Figure 8a,b present the isosurface distributions of TEDM and TMDM for the TMCNB in S_11_. The TEDM density exhibits a decreasing trend along the X, Y and Z directions, while the distribution of TMDM density shows a complementary relationship with TEDM. Specifically, the positive and negative isosurfaces of TEDM are predominantly located at the twisted positions where the three benzene rings are connected. In the X direction, the positive isosurface of TMDM is located above the molecule, and the negative isosurface is positioned above the molecule, while the negative isosurface is located at the lower right corner. In the Y direction, the positive isosurface is distributed in the central region of the long benzene chain, whereas the negative isosurface is distributed along the three shorter benzene chains. In the Z direction, the positive area appears on the left side of the molecule, while the negative area is found on the right side. The separation between the positive and negative TMDM densities is particularly pronounced in the Z direction. For the S_15_ state, the TEDM and TMDM isosurfaces are both small, indicating a weak oscillator strength for this transition. The isosurface distributions of TEDM and TMDM under S_36_ and S_55_ exhibit a similar pattern, with alternating positive and negative regions, as shown in Figures S5–S7. Figures S8 and S9 are the TDM diagrams of TEDM and the TMDM of the TMCNB, and the conclusions obtained are completely consistent with the isosurface diagrams.

Subsequently, a quantitative analysis of the chiral mechanism of the TMCNB was conducted by integrating TEDM and TMDM data. The values of TEDM and TMDM in the four excited states, along with the eigenvalues of the tensor product, are shown in Table 1. These results align precisely with the intensity and direction of the ECD spectrum of the TMCNB, providing strong evidence that the chiral mechanism inferred from TEDM and TMDM is both comprehensive and self-consistent.

#### 2.1.4. Electrostatic Potential and Weak Interaction

Interaction Region Indicator (IRI) [28] can clearly reveal the occurrence and types of chemical bonds and weak interactions between atoms.

The definition of the IRI function is as follows:

(4) $$IRI(r)=\frac{\left|\nabla \rho (r)\right|}{{\left[\rho (r)\right]}^{a}}$$

**Figure 9:**
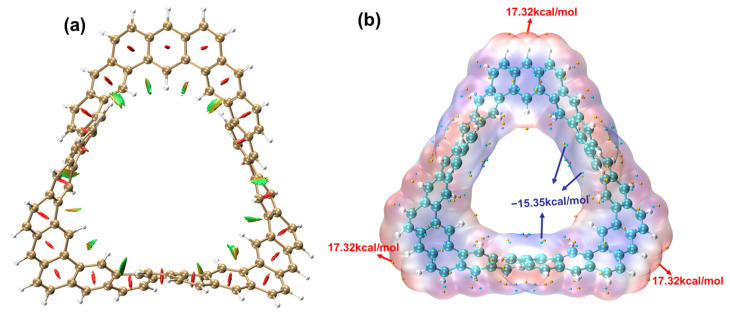
IRI isosurface diagram (**a**) and ESP isosurface diagram (**b**) of TMCNB. In the ESP isosurface diagtam, the blue value is the minimum value and the red value is the maximum value.

Figure 9a presents the IRI isosurface of the TMCNB. This isosurface does not represent the bonding region but only shows the weak interaction region. When the color of the isosurface is red, it indicates that there is a steric hindrance effect at this position. Thus, it can be inferred that there is a steric hindrance effect at the center of each benzene ring. If the isosurface color is green, it indicates van der Waals interactions in that region. Based on this, it can be inferred that there is some steric hindrance within the inner C rings of the TMCNB, while van der Waals interactions dominate between the H atoms.

The electrostatic potential is of great significance in studying intermolecular electrostatic interactions, predicting reactive sites and forecasting molecular properties. The electrostatic potential, V(r), at any given point, r, is represented as the sum of the potentials generated by the nuclear charges and the electron cloud within the molecule:

(5) $$V(r)=\sum_{A}\frac{Z_{A}}{\left|r-R_{A}\right|}-\int \frac{\rho (r’)}{\left|r-r’\right|}dr’$$

In the formula, the first term represents the Coulomb potential generated by the positively charged atomic nuclei, while the second term represents the Coulomb potential produced by the negatively charged electron cloud distribution. Figure 9b is the ESP isosurface diagram of the TMCNB. The ESP can be used to determine the weak interaction of the TMCNB with other molecules and can intuitively understand under what kind of charge the molecule tends to combine with substances. Among them, red represents a positive ESP and blue represents a negative ESP. The yellow dot represents the maximum value and the blue dot represents the minimum value. The ESP around the carbon atom is negative, with a minimum value of −15.35 kcal/mol, which means that this area is prone to electrophilic reactions. While the ESP around the hydrogen atom is positive, with a maximum value of 17.32 kcal/mol, which means that this area is prone to nucleophilic reactions.

#### 2.1.5. Study on Electronic Delocalization and Aromaticity of TMCNB

**Figure 10:**
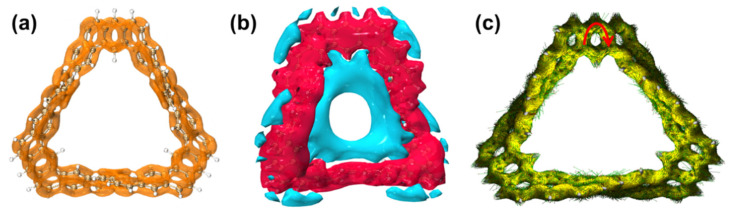
LOL-π isosurface (**a**), ICSSzz isosurface (**b**) and AICD isosurface (**c**) diagrams of TMCNB. The red arrow represents the current direction.

A localized orbital locator (LOL) is a real-space function that can be used to investigate the electronic delocalization path. LOLs can be further decomposed into LOL-π, which contributes to π electrons, to investigate the delocalization path and aromaticity of π electrons in a system. Figure 10a is the LOL-π isosurface diagram of the TMCNB. It can be found that the TMCNB shows a good global delocalization channel and has strong delocalization, which confirms that TMCNB is a fully conjugated structure with a single π surface (π^out^). The iso-chemical shielding surface (ICSS) is a real-space function closely related to the nucleus-independent chemical shift (NICS), which can clearly reveal the degree of magnetic shielding and deshielding effects of delocalized electrons in different regions and can be used to study the aromaticity of cyclic molecules. The red (blue) contour surface in Figure 10b represents the region that generates 5 ppm shielding (deshielding) for the external magnetic field in the Z direction. Through analysis, it is found that the main stem of the TMCNB is red, which belongs to the shielding area, while its surroundings and interior are deshielding areas. Through calculation, the ICSSZZ value at 1 Å above the molecular plane of the TMCNB is 45.8 ppm, which indicates that the TMCNB has aromaticity, and its aromaticity is mainly contributed by π^out^. Figure 10c is the anisotropy of the induced current density (AICD) of the TMCNB, which shows the direction of the annular induced current under the action of an external magnetic field. The circular current following the left-hand rule indicates its aromaticity. The circular currents on the benzene ring in the figure are all clockwise, so it has aromaticity. However, in the overall macrocycle, although there is a complete electronic delocalization channel, the direction is irregular, so the TMCNB has local aromaticity.

### 2.2. Photoelectric Properties and Physical Mechanism of MCNB2—A Designed Isomer of TMCNB

#### 2.2.1. Structure and Properties of MSNB2

**Figure 11:**
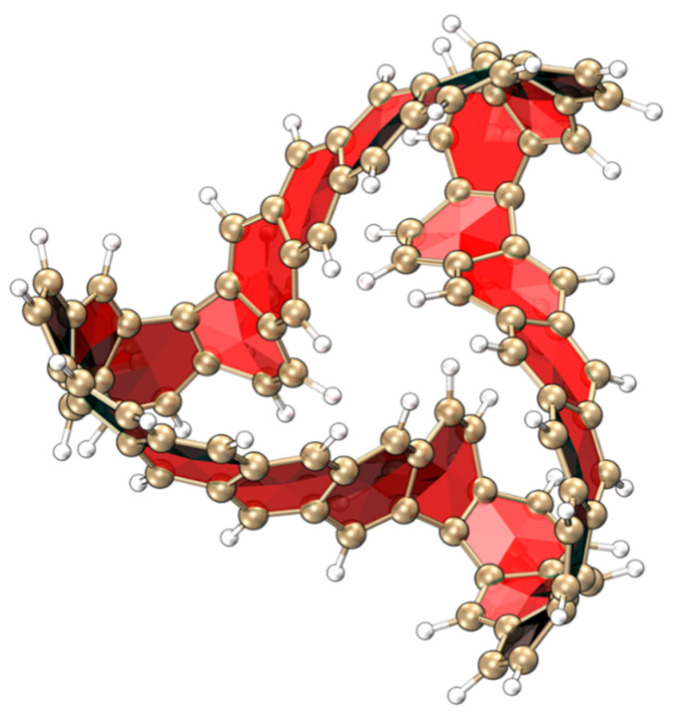
3D structure diagram of MCNB2.

Figure 11 shows the 3D structure of MCNB2. This structure is a windmill-shaped structure obtained by twisting each benzene ring chain in the TMCNB structure. Different structural configurations often lead to different optical properties. Studying this structure–activity relationship is of great significance for in-depth understanding of the properties and functions of molecules, developing new compounds, and optimizing the performance of existing compounds. The isomer of the TMCNB, MCNB2, was designed. In view of this, this section will discuss research on the physical properties of MCNB2.

**Figure 12:**
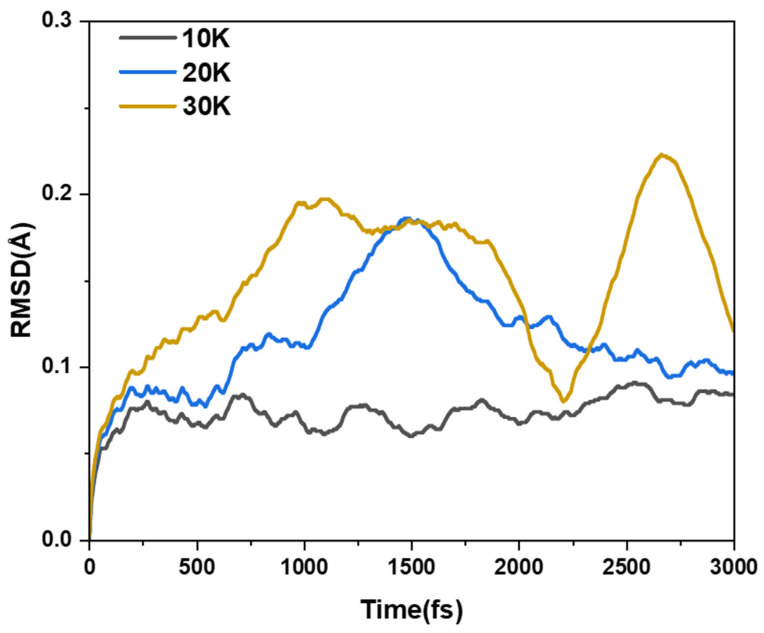
RMSD diagrams of MCNB2 at temperatures of 10 K 20 K and 30 K.

First of all, we discuss the thermal stability of MCNB2 through ab initio molecular dynamics calculations. Thus, the temperature conditions under which it can stably exist are obtained. Therefore, we simulated the movement trajectories at different temperatures in a vacuum for 3000 fs and simulated the root mean square deviation (RMSD) of their ab initio dynamic trajectories relative to the optimized structure. Figure 12 shows the RMSD diagrams at 10 K, 20 K and 30 K, respectively. At a temperature of 10 K, the RMSD curve has a certain periodicity. While at temperatures of 20 K and 30 K, although the curve does not have a clear periodicity, there are peaks, indicating that the structure is relatively stable at these temperatures. When the temperature reaches 40 K, the RMSD curve keeps rising within 3000 fs, indicating that the stability of this structure is very poor at a temperature of 40 K. We also calculated the RMSD curves at 50 K and 60 K. The results show that as the temperature increases, the stability of the structure is also worse (Figure S10). Then, we plotted the superposition of the motion trajectories of MCNB2 at 10–60 K. One frame structure is obtained every 100 fs from the AIMD trajectory, with a total of 30 frames. The atom color from red to blue represents the position of the atom during the simulation time from 0 K to 3000 fs, as shown in Figure S11. It can be found that the movement amplitude of MCNB2 at temperatures of 10–30 K is relatively small, and the structure is in a stable state. When the temperature rises, the movement becomes more intense, especially at 50 K and 60 K, the movement trajectory is quite intense, and at 60 K, the structure even undergoes severe deformation.

#### 2.2.2. HOMO–LUMO Molecular Orbital Analysis

**Figure 13:**
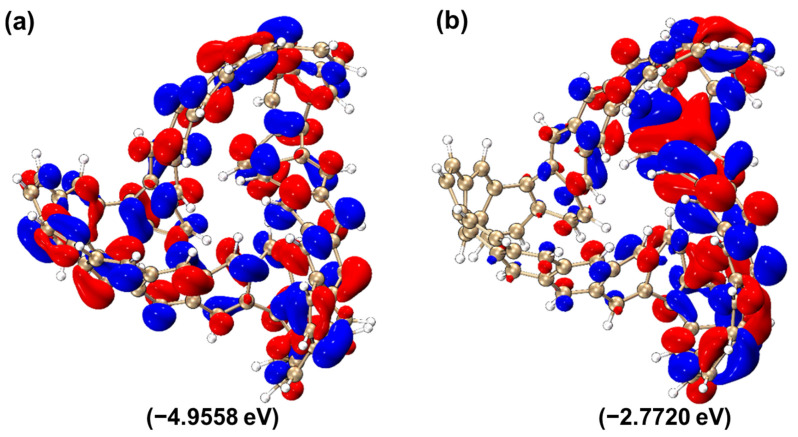
HOMO (**a**) and LUMO (**b**) isosurface diagram of MCNB2.

As shown in Figure 13, the HOMO and LUMO isosurfaces of MCNB2 are presented. Among them, the HOMO energy level is −4.9558 eV, the LUMO energy level is −2.7720 eV and the HOMO–LUMO gap is 2.1839 eV. It is worth noting that the HOMO–LUMO gap value of MCNB2 is smaller than that of the TMCNB. Based on this, it can be inferred that MCNB2 is more likely to undergo transitions compared to the TMCNB. In the isosurface diagrams of HOMO and LUMO, electrons and holes are evenly distributed, and this feature reflects the characteristics of local excitation. In addition, the electron transition in LUMO mainly occurs on the right side of the molecule, and MCNB2 also shows the characteristics of π excitation.

#### 2.2.3. OPA and TPA Characteristics

**Figure 14:**
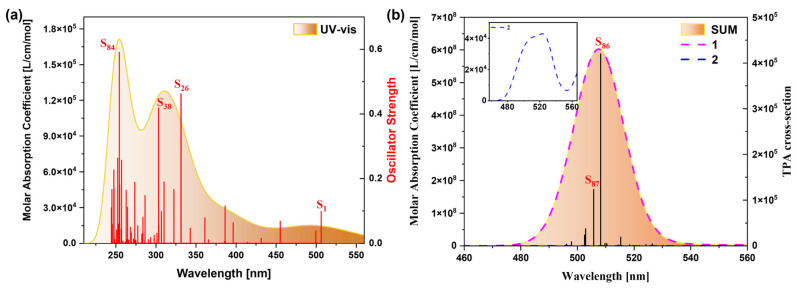
OPA spectrum (**a**) and TPA spectrum (**b**) of MCNB2.

Figure 14a shows the single-photon absorption spectrum of MCNB2, whose spectral range is in the range of 210–560 nm, covering part of the ultraviolet and visible light regions. There are three absorption peaks in this spectrum, among which the absorption peak at 254.79 nm has the strongest intensity and is mainly contributed to by the excited state S_84_. Figure 14b is the two-photon absorption spectrum of MCNB2, with the spectral region in the range of 480–560 nm. Its absorption peaks are mainly contributed to by the excited states S_86_ and S_87_, and the two-photon spectrum is in the visible light region.

**Figure 15:**
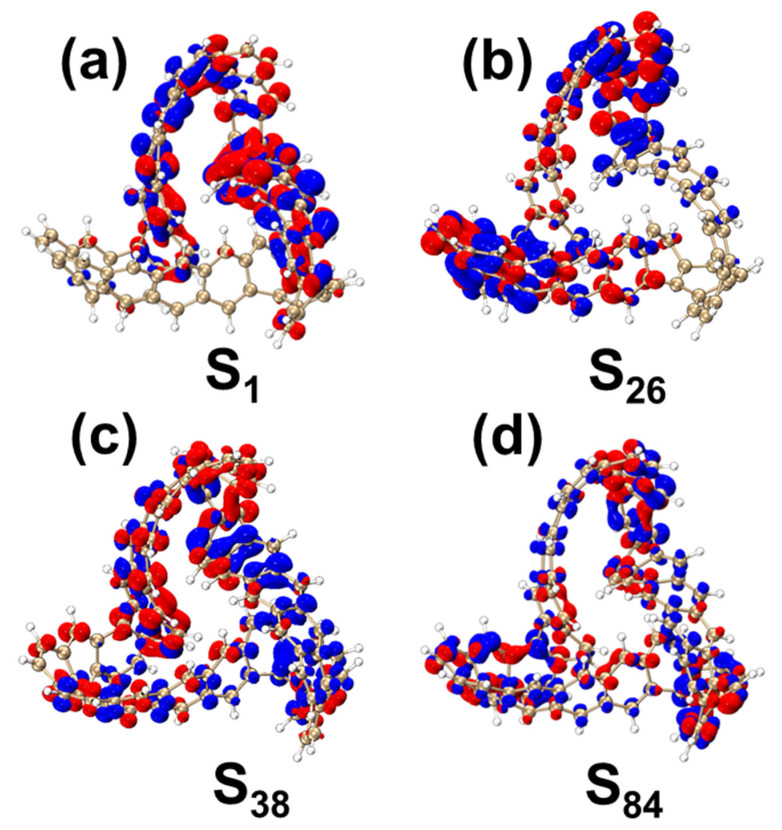
CDD diagrams of MCNB2 under S_1_ (**a**), S_26_ (**b**), S_38_ (**c**) and S_84_ (**d**). In the CDD diagram, blue represents holes and red represents electrons. The isovalue is 0.005.

Figure 15a and Figure S12a present the CDD and TDM diagram of MCNB2 in the S_1_, showing that electrons and holes are primarily localized on the right side of the molecule. Figure 15b and Figure S12b are the CDD and TDM diagram for the excited state S_14_, where electrons and holes are more evenly distributed on the left side of the molecule. In both of these excited states, the excitations are classified as local excitations. In the CDD diagram in the S_26_, electrons are predominantly located on the left side of MCNB2, while holes are positioned in the lower right corner, indicating the presence of charge transfer excitation. The TDM diagram reveals distinct bright regions along the diagonal, with additional bright areas on either side, suggesting a combination of local excitation and electron transfer excitation, as seen in Figure 15c and Figure S12c. In the S_84_, electrons and holes are distributed across the entire structure, with multiple separated regions for both, indicating a local excitation accompanied by charge transfer, as seen in Figure 15d and Figure S12d.

**Figure 16:**
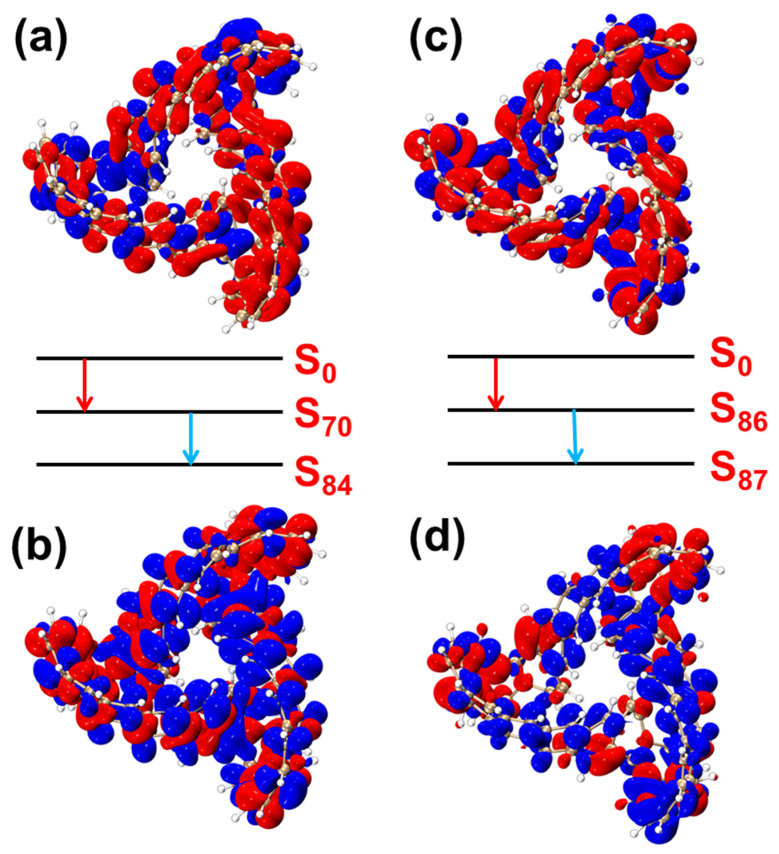
Electron–hole pair density of the two-step transition of TMCNB in S_84_, from the ground state to the intermediate state (**a**) and from the intermediate state to the final state (**b**). Electron–hole pair density of the two-step transition of TMCNB in S_87_, from the ground state to the intermediate state (**c**) and from the intermediate state to the final state (**d**). In the electron-hole pair density diagram, blue represents holes and red represents electrons. The red arrows represent the first step and the blue arrows represent the second step. The isovalue is 0.0006.

Figure 16 presents the TDM diagrams and electron–hole density diagrams for the two-step transitions of the excited states that significantly contribute to the two-photon absorption peak of MCNB2. Among them, Figure 16a,b depict the two-step transition process of S_0_–S_86_. The initial transition, S_0_→S_84_, shows a prominent bright region along the main diagonal in the TDM diagram, indicating local excitation, as confirmed by the electron–hole density analysis (Figure 16c). In the second transition process, S_84_→S_86_, the separation between electrons and holes is substantial, with electrons predominantly located at the top of the molecule and holes in the central region, highlighting clear charge-transfer excitation. This charge-transfer nature is further supported by the bright regions outside the main diagonal in the TDM diagram. Figure 16c,d present the two-step transition process of S_0_–S_87_. The transition from S_0_ to S_70_ exhibits a combination of local excitation and charge transfer, with alternating electron and hole isosurfaces. However, the isosurface of holes is more than that of electrons. It can be inferred that electrons have transitioned to the area outside the molecule. The transition from S_70_ to S_87_ is primarily characterized by charge transfer, with a higher electron density and fewer holes. The corresponding TDM diagrams are provided in Figure S13.

#### 2.2.4. The Chiral Physical Mechanism of the Electronic Circular Dichroism of MCNB2

**Figure 17:**
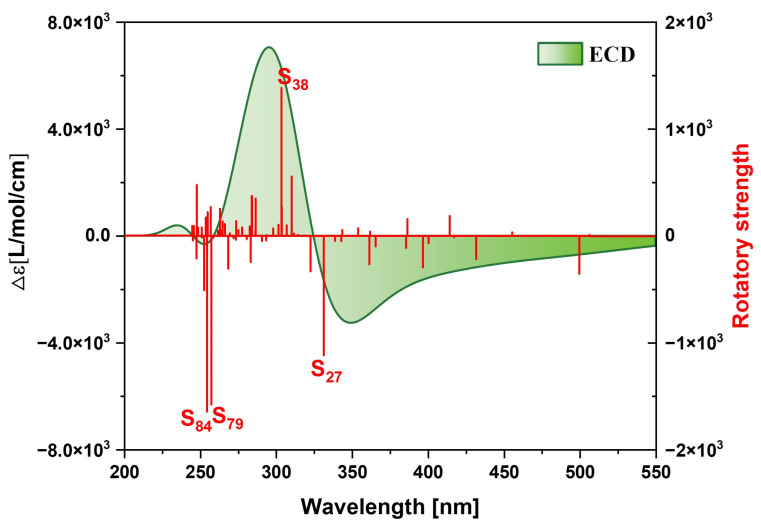
The ECD spectrum of MCNB2.

**Figure 18:**
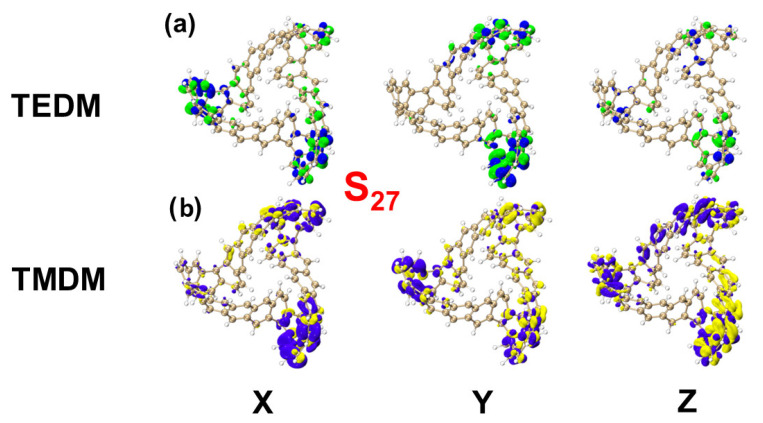
The TEDM/TMDM isosurfaces of MCNB2 in the excited states of S_27_ (**a**,**b**). The blue and green isosurfaces represent the positive and negative TEDM, respectively, and the yellow and purple isosurfaces represent the positive and negative TMDM, respectively.

Figure 17 shows the ECD spectrum of MCNB2, which features two positive and two negative absorption peaks.. After analysis, the excited states that contribute significantly to these peaks are identified as S_27_, S_38_, S_79_ and S_84_. Similar to the case of TMCNB, the TEDM density of MCNB2 decreases in the X, Y and Z directions, while the TMDM density gradually increases. The TEDM and TMDM isosurface regions are primarily concentrated at the three molecular tips, with distinct separation in both the positive and negative regions of TEDM and TMDM, which induces chirality, as shown in Figure 18 and Figure S14–S16. Figures S17 and S18 are the TDM diagrams of TEDM and TMDM of MCNB2, with the findings aligning well with the isosurface analysis. The TEDM and TMDM values for the four excited states of MCNB2, along with the eigenvalues of the tensor product, are summarized in Table 2. These results closely match the intensity and direction of the ECD spectrum, providing strong support for the interpretation of the chiral mechanism.

#### 2.2.5. Study on Electronic Delocalization and Aromaticity of MCNB2

**Figure 19:**
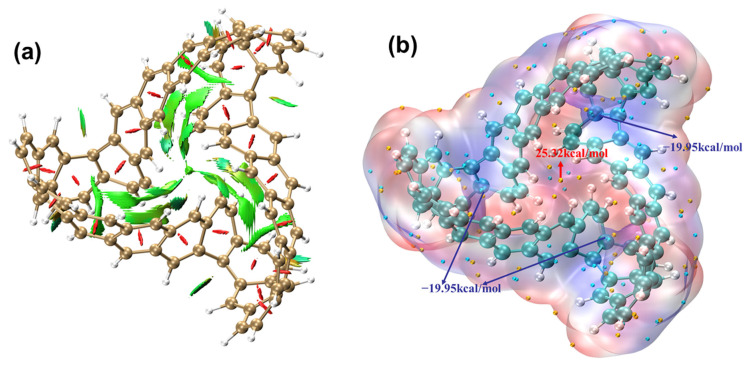
The IRI isosurface diagram (**a**) and ESP isosurface diagram (**b**) of MCNB2. In the ESP isosurface diagtam, the blue value is the minimum value and the red value is the maximum value.

Figure 19a presents the IRI isosurface of MCNB2. The analysis shows that there is a steric hindrance effect at the center of each benzene ring. Additionally, the structural center of MCNB2, as well as the hydrogen bonds between the tips and the long chains of MCNB2, exhibit strong van der Waals interactions. Figure 19b is the ESP isosurface diagram of MCNB2. Among them, the ESP around the carbon atom is negative, with a minimum value of −19.95 kcal/mol, which means that this area is prone to electrophilic reactions. While the ESP around the hydrogen atom is positive, the maximum point is located at the center of the molecule, with a value of 25.32 kcal/mol, and this area is prone to nucleophilic reactions.

**Figure 20:**
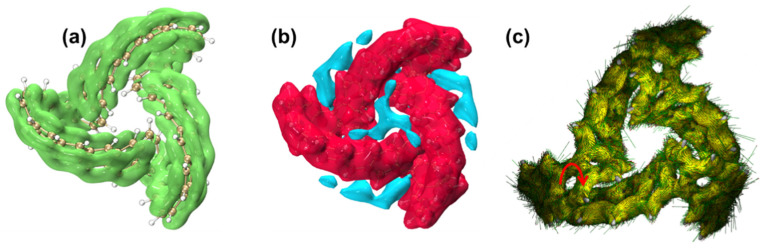
LOL isosurface (**a**), ICSSzz isosurface (**b**) and AICD isosurface (**c**) diagrams of MCNB2. The red arrow represents the current direction.

Figure 20a–c show the LOL-π isosurface diagram, ICSSzz isosurface diagram and AICD isosurface diagram of MCNB2, respectively. The analysis found that there is a good global delocalization channel in LOL-π, indicating that MCNB2 has strong delocalization. The main stem area of MCNB2 is a shielding area, while its surroundings and interior are deshielding areas. The ICSS_ZZ_ value at 1 Å above the molecular plane of MCNB2 is 35.2 ppm. This result indicates that MCNB2 has π^out^ aromaticity. In addition, the ring current on the benzene ring follows the left-hand rule, further confirming that MCNB2 has local aromaticity.

## 3. Computational Methods

This work is based on the DFT calculation method, the B3LYP functional [29] and the 6–31G (d, p) basis set [30] combined with the DFT-d3 correction [31], and Gaussian software [32] was used to calculate the molecular structure. The electronic excitation calculation is based on the TD-DFT calculation method, the CAM-B3LYP functional [33] and the 6–311G (d, p) basis set. The magnetic induction current density was calculated using AICD 2.0 software. The Multiwfn program [34] was used to analyze the wave functions including TEDM\TMDM [35,36], electrostatic potential (ESP) [37], ICSS, LOL, IRI and charge density difference (CDD) [38]. All 3D graphs, except for the magnetic induction current density, were plotted using the VMD program [39]. The construction of MCNB2 was accomplished using the chemdraw 3D program. Molecular dynamics simulation of MCNB2 was conducted using the cp2k program [40], and visualization analysis was carried out using the VMD and origin programs. Since the solvent effect is not considered in this work, all calculations were performed under vacuum conditions.

## 4. Conclusions

This paper presents an in-depth study, based on density functional theory, of the single-photon and two-photon absorption properties, electronic circular dichroism, and aromaticity of the TMCNB, as well as the underlying physical mechanisms. Through wave function analysis, the charge transfer mechanism, chirality, and delocalization are discussed. CDD and TDM are used to describe the one-photon electron transition mechanism of the TMCNB, revealing that the transition mode is predominantly local excitation, while for two-photon transitions, it is determined through research that they are jointly affected by local excitation and charge transfer. TEDM and TMDM are used to explain the chiral mechanism of the TMCNB. Additionally, we examine the weak interactions within the structure, identifying a dispersion effect between hydrogen bonds. Finally, using a LOL and the ICSS and AICD, we investigate the aromaticity of the TMCNB, finding that it exhibits local π^out^ aromaticity. Taking the TMCNB structure as the prototype, we reconstructed the new structure, MCNB2, and studied its above-mentioned properties. The results show that compared with the TMCNB, MCNB2 exhibits a higher propensity for electronic transitions, though its chirality and aromaticity are reduced. This study not only provides valuable insights for the practical application of Möbius carbon nanoring materials in optoelectronic fields, but also offers guidance for the design and construction of similar materials.

## Figures and Tables

**Table 1:** The value of the transition electric/magnetic dipole moment and the eigenvalue of their tensor product of TMCNB.

		X	Y	Z	Eigenvalue
S_11_	TEDM	2.6963	5.8930	0.0002	13.82759357
	TMDM	−0.8881	−1.9401	−0.0012	
S_15_	TEDM	−0.0177	0.0323	−0.0001	0.00170296
	TMDM	0.0203	−0.0416	−0.0003	
S_36_	TEDM	1.6935	−1.2341	−0.0006	−5.15281076
	TMDM	1.9867	−1.4491	0.0000	
S_55_	TEDM	0.0017	0.0005	2.7348	−12.73021732
	TMDM	−0.0020	0.0004	4.6549	

**Table 2:** The value of the transition electric/magnetic dipole moment and the eigenvalue of their tensor product of MCNB2.

		X	Y	Z	Eigenvalue
S_27_	TEDM	−1.0245	−1.9976	0.0010	−4.69866582
	TMDM	−4.3178	−0.1377	0.0102	
S_38_	TEDM	1.1735	1.6742	0.0127	5.95234577
	TMDM	−2.7698	−1.6137	−0.0259	
S_79_	TEDM	−0.0053	−0.0070	−1.4730	−6.71631021
	TMDM	0.0063	0.0135	−4.5597	
S_84_	TEDM	0.0121	0.0223	−2.2231	−6.98005882
	TMDM	−0.0225	−0.0091	−3.1400	

## Data Availability

Data are contained within the article and Supplementary Materials.

## Supplementary Materials

The following supporting information can be downloaded at: https://www.mdpi.com/article/10.3390/molecules29194621/s1, Figure S1: UV-Vis spectrum of triply twisted Möbius carbon nanobelt; Figure S2: TDM diagrams of TMCNB under S_11_ (a), S_36_ (b), S_55_ (c), S_63_ (d); Figure S3: TDM of the two-step transition of TMCNB in S_29_, from the ground state to the intermediate state (a) and from the intermediate state to the final state (b). TDM of the two-step transition of TMCNB in S_46_, from the ground state to the intermediate state (c) and from the intermediate state to the final state (d); Figure S4: TDM of the two-step transition of TMCNB in S_61_, from the ground state to the intermediate state (a) and from the intermediate state to the final state (b). TDM of the two-step transition of TMCNB in S_89_, from the ground state to the intermediate state (c) and from the intermediate state to the final state (d); Figure S5: Isosurfaces of TEDM/TMDM of TMCNB in the excited states of S_15_ (a,b). The blue and green isosurfaces represent the positive and negative TEDM, respectively, and the yellow and purple isosurfaces represent the positive and negative TMDM, respectively; Figure S6: Isosurfaces of TEDM/TMDM of TMCNB in the excited states of S_36_ (a,b). The blue and green isosurfaces represent the positive and negative TEDM, respectively, and the yellow and purple isosurfaces represent the positive and negative TMDM, respectively; Figure S7: Isosurfaces of TEDM/TMDM of TMCNB in the excited states of S_55_ (a,b). The blue and green isosurfaces represent the positive and negative TEDM, respectively, and the yellow and purple isosurfaces represent the positive and negative TMDM, respectively; Figure S8: TDM diagrams of TEDM/TMDM of TMCNB under S_11_ and S_15_; Figure S9: TDM diagrams of TEDM/TMDM of TMCNB under S_36_ and S_55_; Figure S10: RMSD diagrams of MCNB2 at temperatures of 40 K, 50 K, and 60 K; Figure S11: The AIMD trajectory of MCNB2 at 10–60 K. One frame structure is taken every 100 frames, with a total of 30 frames. Red and blue represent the structures in the early and late stages of the simulation respectively; Figure S12: TDM diagrams of MCNB2 under S_1_ (a), S_14_ (b), S_26_ (c), S_84_ (d); Figure S13: TDM of the two-step transition of TMCNB in S_84_, from the ground state to the intermediate state (a) and from the intermediate state to the final state (b). TDM of the two-step transition of TMCNB in S_87_, from the ground state to the intermediate state (c) and from the intermediate state to the final state (d); Figure S14: Isosurfaces of TEDM/TMDM of TMCNB in the excited states of S_38_ (a,b). The blue and green isosurfaces represent the positive and negative TEDM, respectively, and the yellow and purple isosurfaces represent the positive and negative TMDM, respectively; Figure S15: Isosurfaces of TEDM/TMDM of TMCNB in the excited states of S_79_ (a,b). The blue and green isosurfaces represent the positive and negative TEDM, respectively, and the yellow and purple isosurfaces represent the positive and negative TMDM, respectively; Figure S16: Isosurfaces of TEDM/TMDM of TMCNB in the excited states of S_84_ (a,b). The blue and green isosurfaces represent the positive and negative TEDM, respectively, and the yellow and purple isosurfaces represent the positive and negative TMDM, respectively; Figure S17: TDM diagrams of TEDM/TMDM of MCNB2 under S_27_ and S_38_; Figure S18: TDM diagrams of TEDM/TMDM of MCNB2 under S_79_ and S_84_.
