# Supplementary material for: Optoelectronic Properties of Triply Twisted Möbius Carbon Nanobelt and the Design of Its Isomeric Nanomaterials
Source: Molecules. 2024 Sep 29;29(19):4621. doi: 10.3390/molecules29194621 (PMC11477768; doi:10.3390/molecules29194621)
Supplement: Supplementary file 1 [file molecules-29-04621-s001.zip › molecules-3202903-supplementary.pdf]

Supporting information

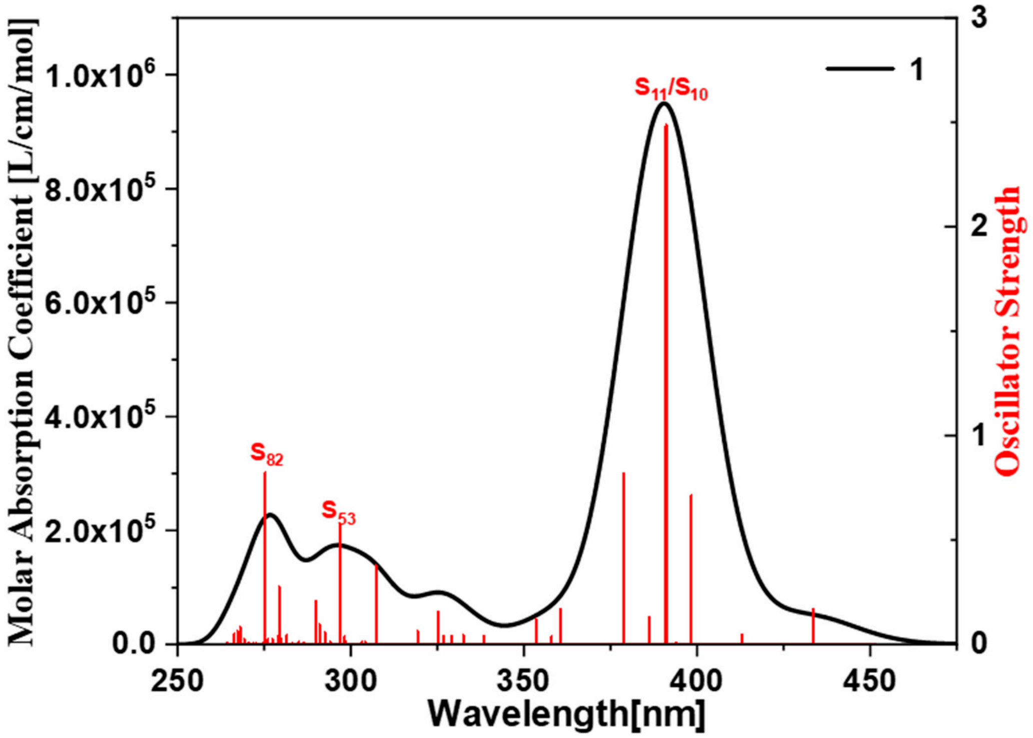

Figure S1. UV-Vis spectrum of triply twisted Möbius carbon nanobelt.

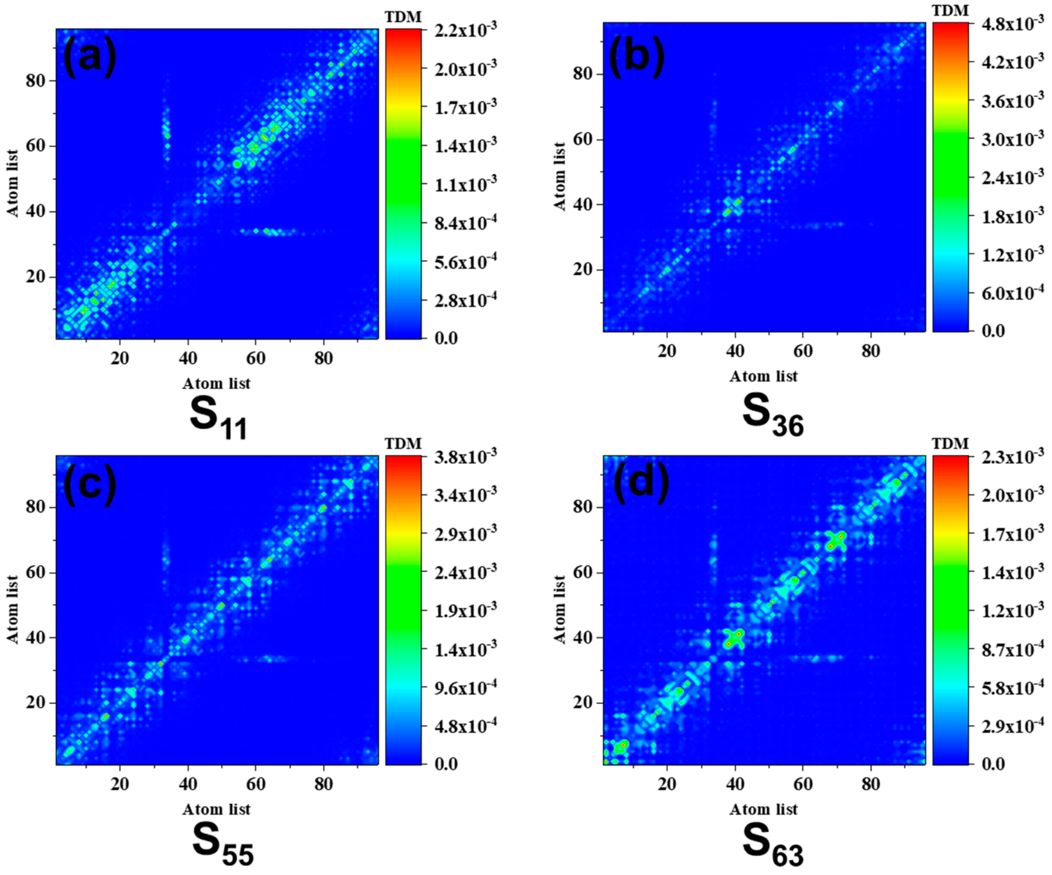

Figure S2. TDM diagrams of TMCNB under S_{11} (a), S_{36} (b), S_{55} (c), S_{63} (d).

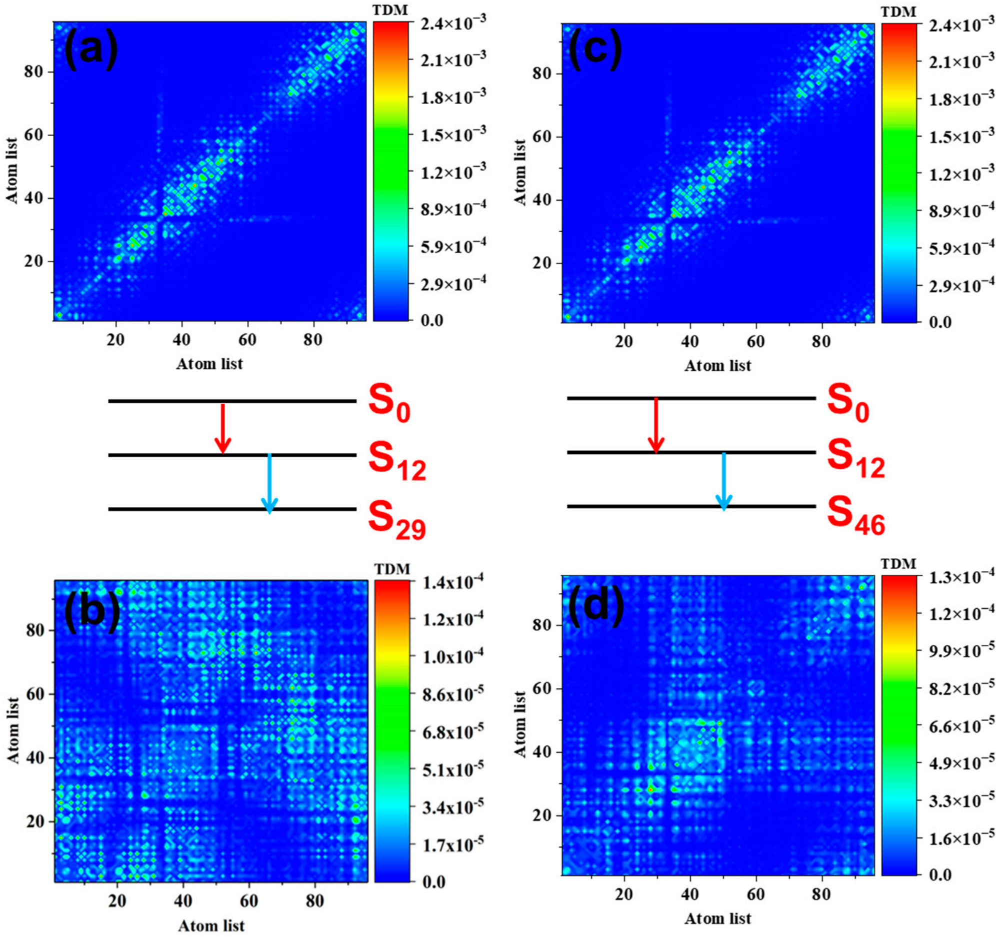

Figure S3. TDM of the two-step transition of TMCNB in S_{29}, from the ground state to the intermediate state (a) and from the intermediate state to the final state (b). TDM of the two-step transition of TMCNB in S_{46}, from the ground state to the intermediate state (c) and from the intermediate state to the final state (d).

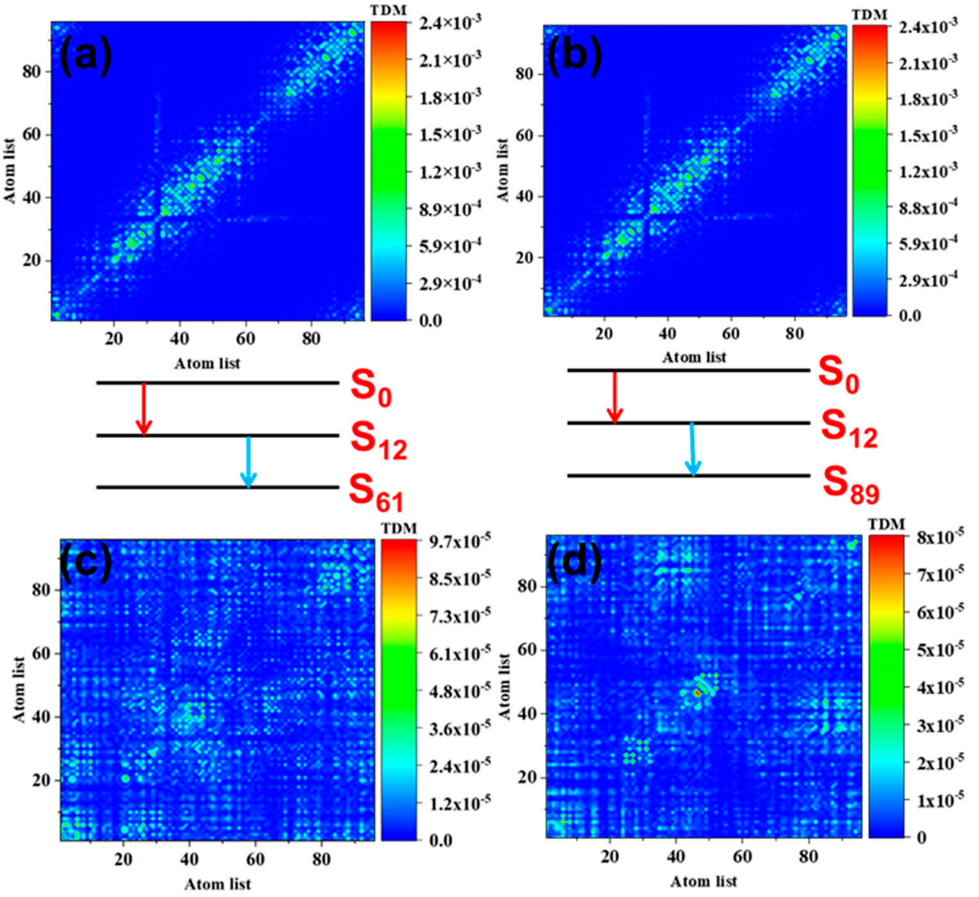

Figure S4. TDM of the two-step transition of TMCNB in S_{61}, from the ground state to the intermediate state (a) and from the intermediate state to the final state (b). TDM of the two-step transition of TMCNB in S_{89}, from the ground state to the intermediate state (c) and from the intermediate state to the final state (d).

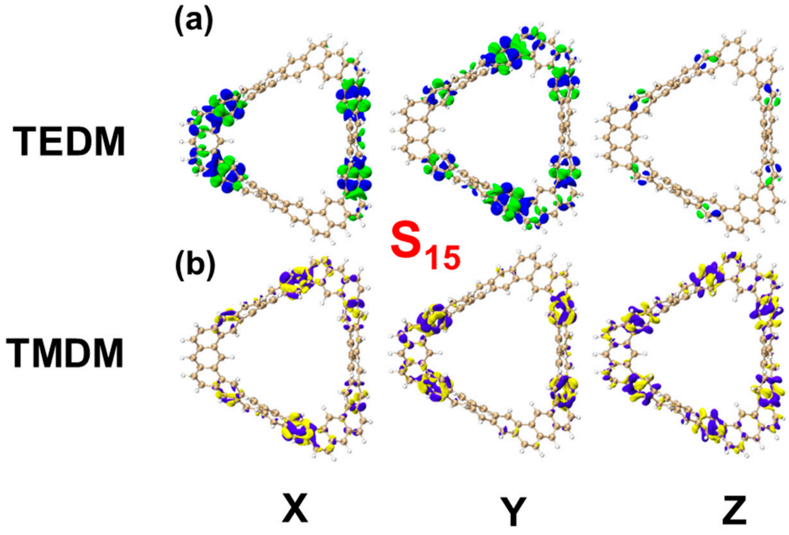

Figure S5. Isosurfaces of TEDM/TMDM of TMCNB in the excited states of S_{15} (a, b). The blue and green isosurfaces represent the positive and negative TEDM, respectively, and the yellow and

purple isosurfaces represent the positive and negative TMDM, respectively.

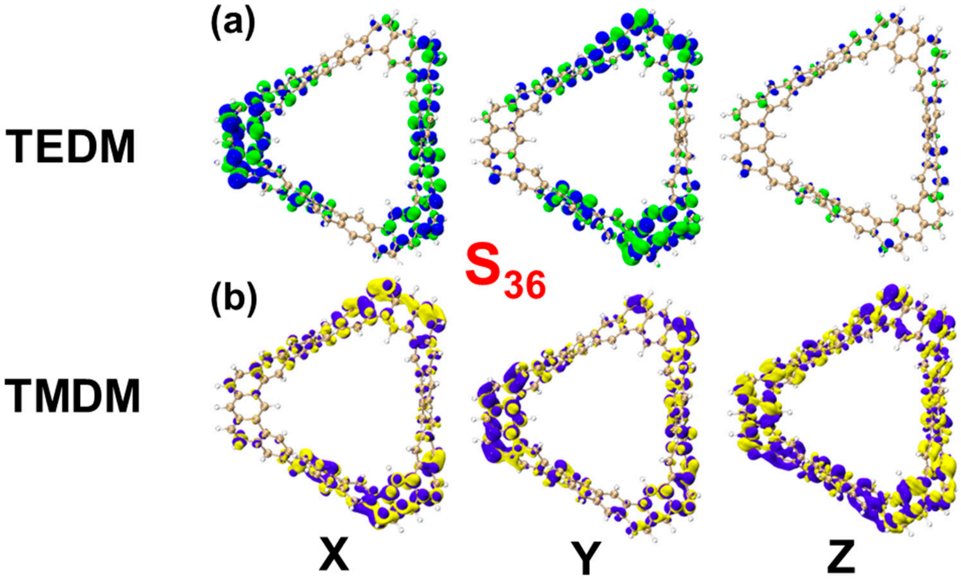

Figure S6. Isosurfaces of TEDM/TMDM of TMCNB in the excited states of S_{36} (a, b). The blue and green isosurfaces represent the positive and negative TEDM, respectively, and the yellow and purple isosurfaces represent the positive and negative TMDM, respectively.

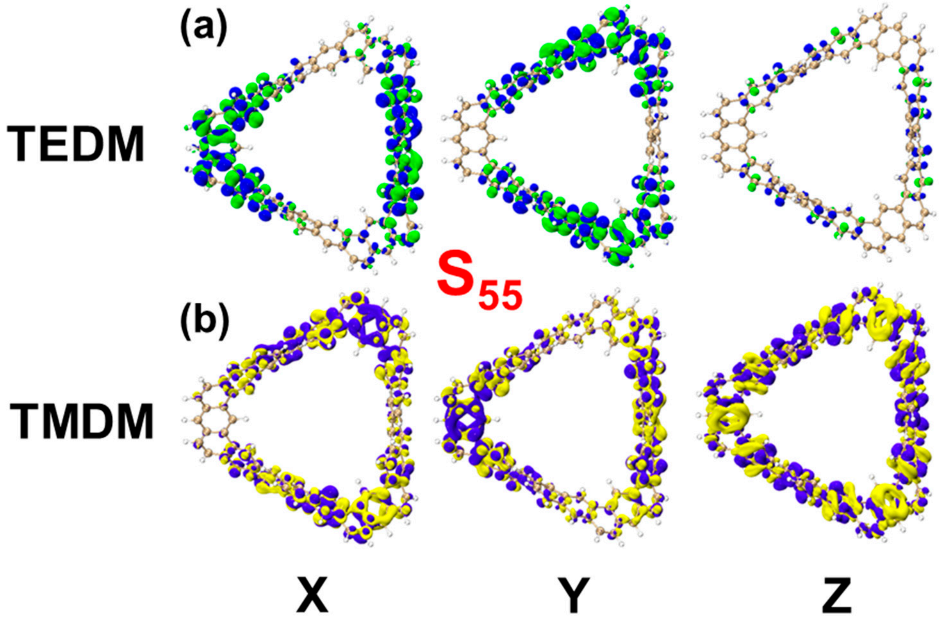

Figure S7. Isosurfaces of TEDM/TMDM of TMCNB in the excited states of S_{55} (a, b). The blue and green isosurfaces represent the positive and negative TEDM, respectively, and the yellow and

purple isosurfaces represent the positive and negative TMDM, respectively.

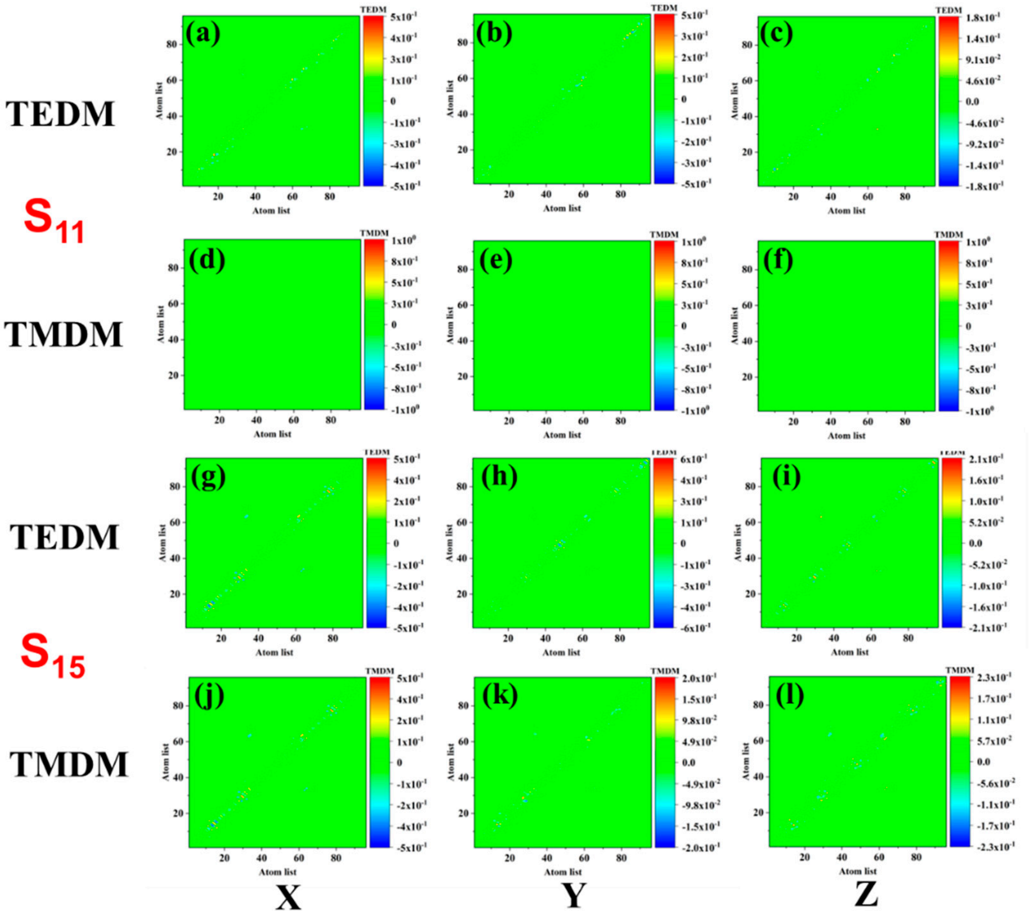

Figure S8. TDM diagrams of TEDM/TMDM of TMCNB under S_{11} and S_{15}.

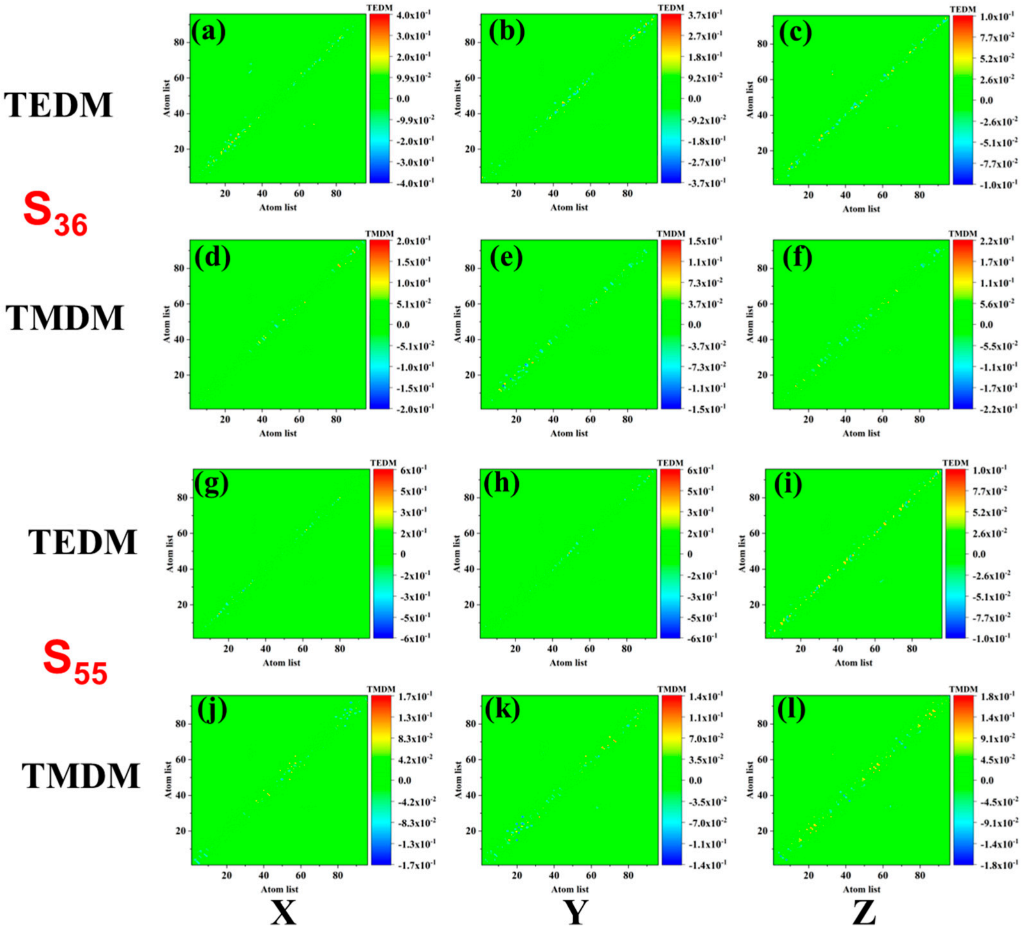

Figure S9. TDM diagrams of TEDM/TMDM of TMCNB under S_{36} and S_{55}.

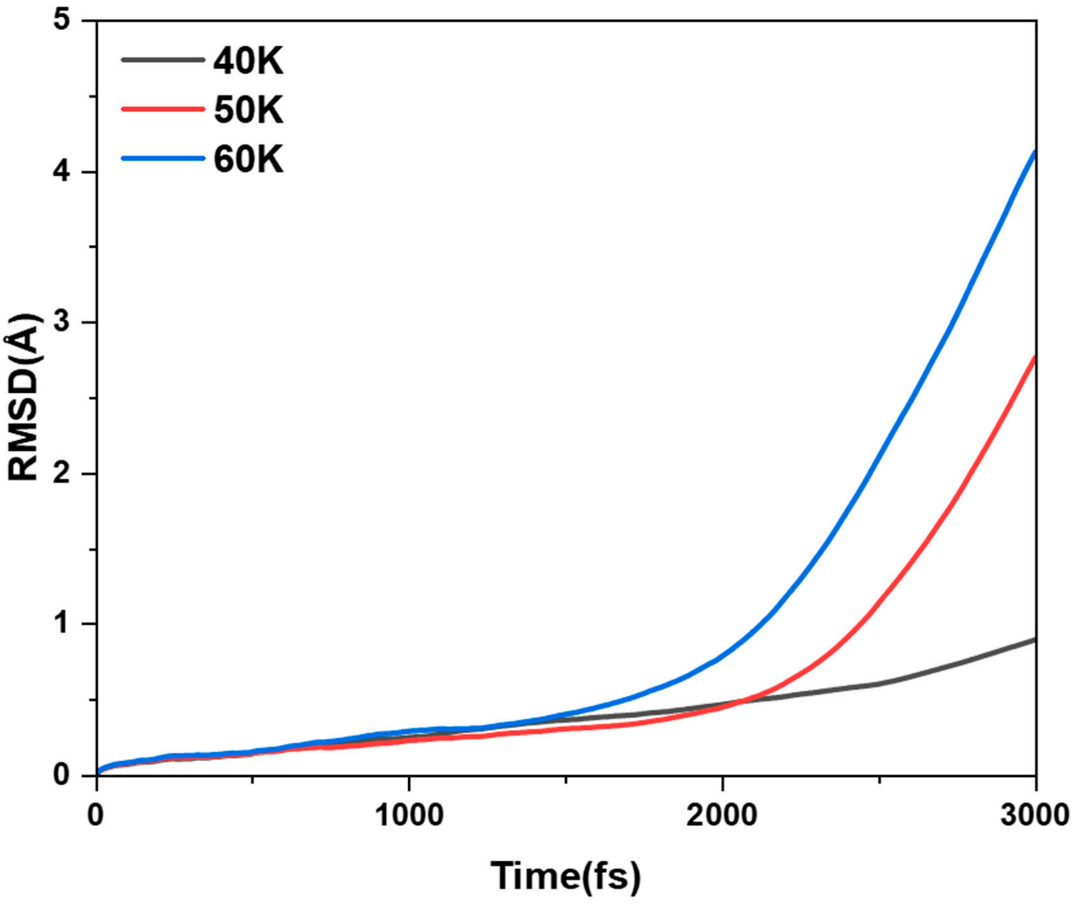

Figure S10. RMSD diagrams of MCNB2 at temperatures of 40k, 50k, and 60k.

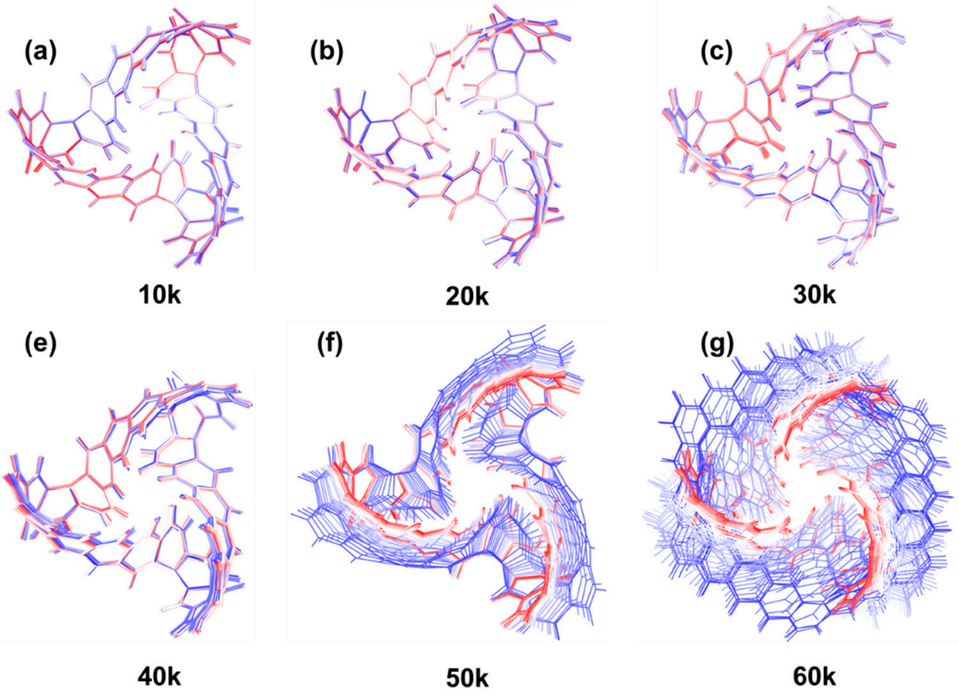

Figure S11. The AIMD trajectory of MCNB2 at 10 - 60K. One frame structure is taken every 100 frames, with a total of 30 frames. Red and blue represent the structures in the early and late stages of the simulation respectively.

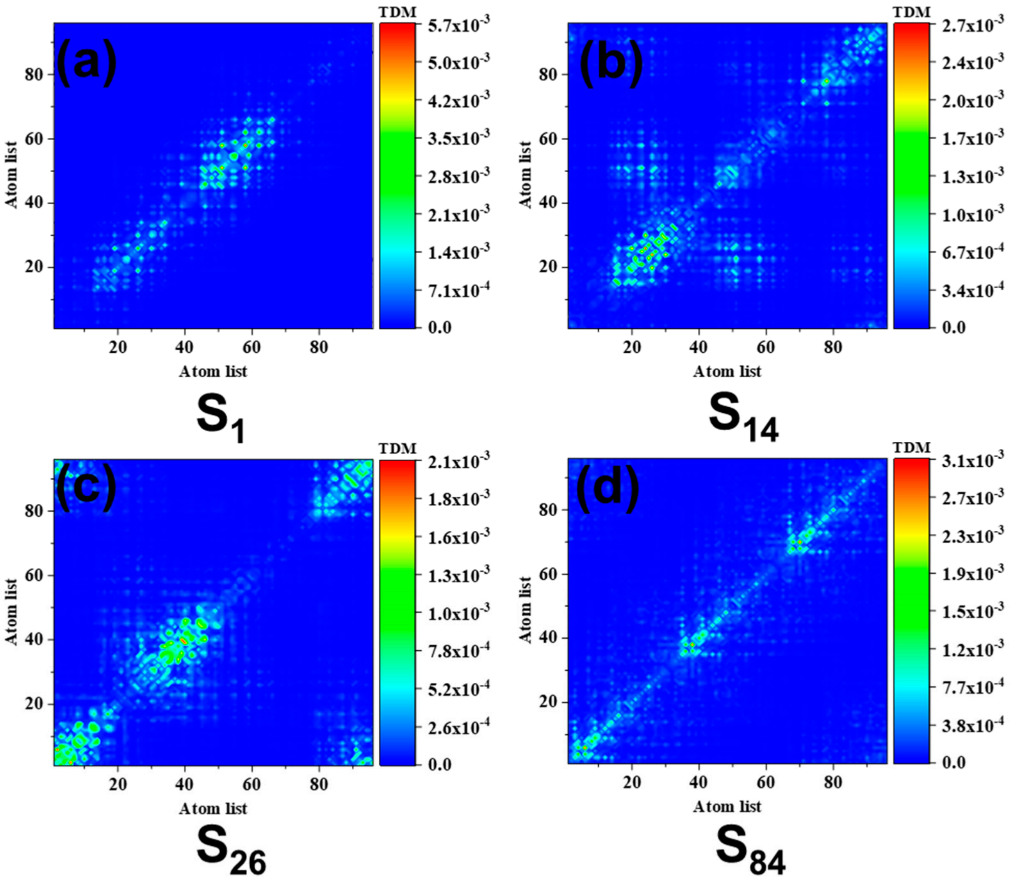

Figure S12. TDM diagrams of MCNB2 under S_1 (a), S_{14} (b), S_{26} (c), S_{84} (d).

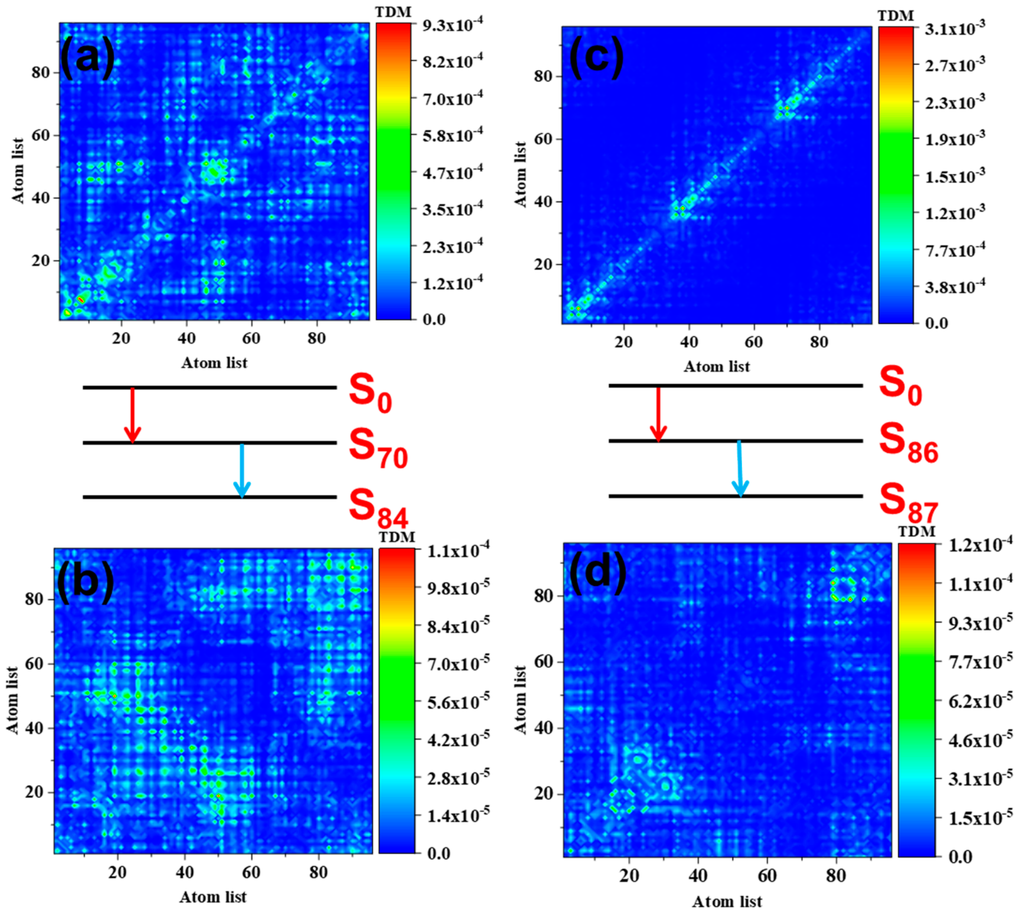

Figure S13. TDM of the two-step transition of TMCNB in S_{84}, from the ground state to the intermediate state (a) and from the intermediate state to the final state (b). TDM of the two-step transition of TMCNB in S_{87}, from the ground state to the intermediate state (c) and from the intermediate state to the final state (d).

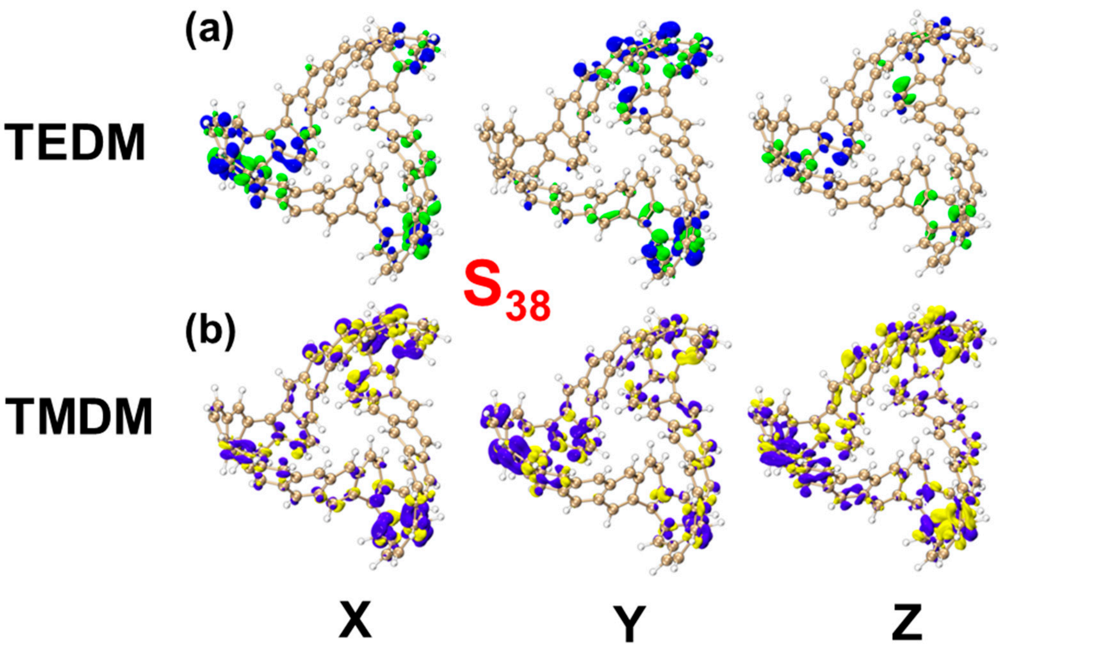

Figure S14. Isosurfaces of TEDM/TMDM of TMCNB in the excited states of S_{38} (a, b). The blue

and green isosurfaces represent the positive and negative TEDM, respectively, and the yellow and purple isosurfaces represent the positive and negative TMDM, respectively.

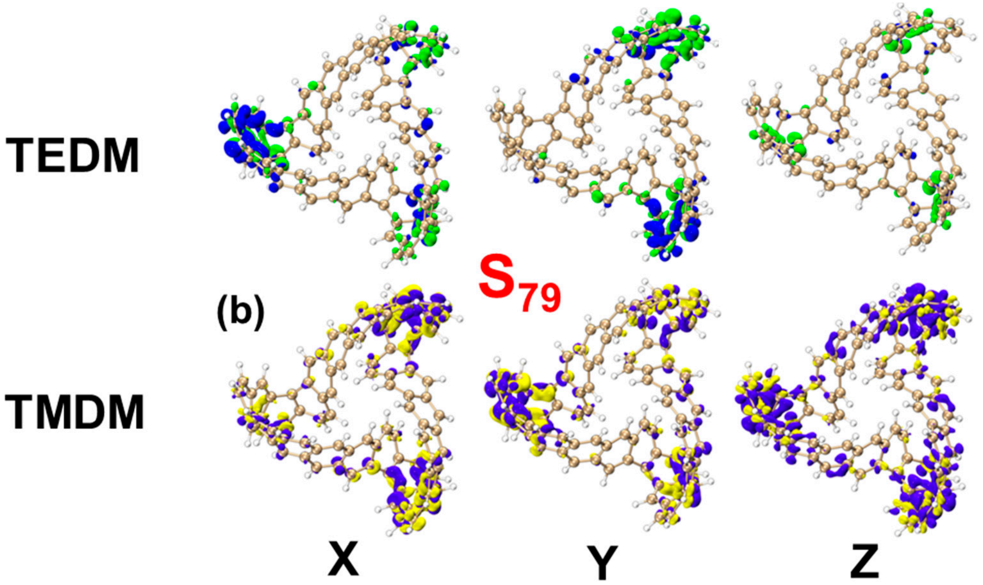

Figure S15. Isosurfaces of TEDM/TMDM of TMCNB in the excited states of S_{79} (a, b). The blue and green isosurfaces represent the positive and negative TEDM, respectively, and the yellow and purple isosurfaces represent the positive and negative TMDM, respectively.

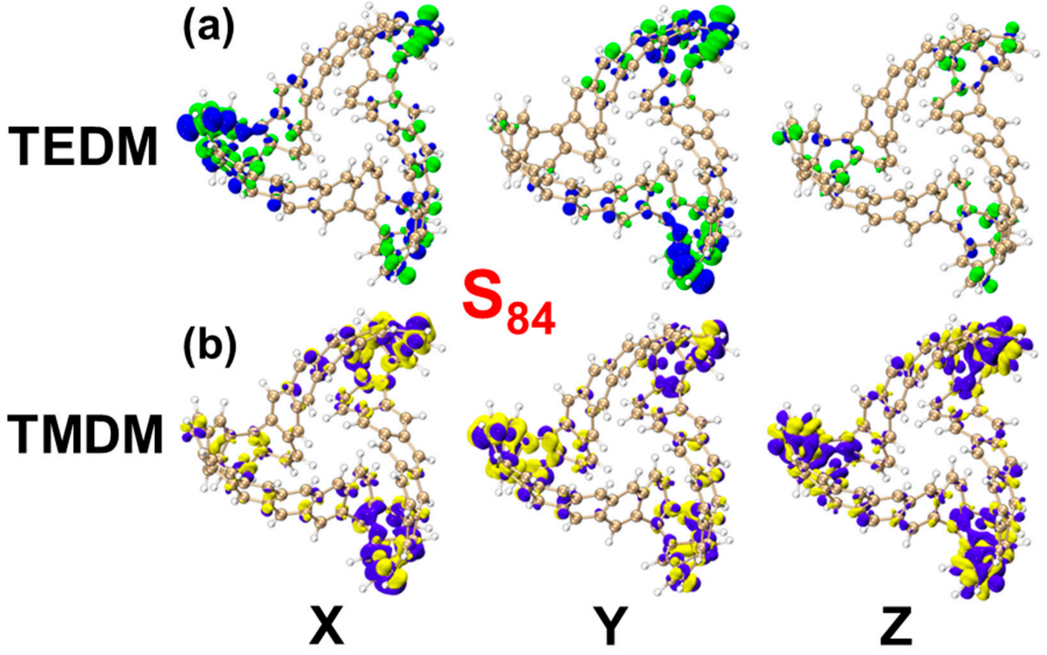

Figure S16. Isosurfaces of TEDM/TMDM of TMCNB in the excited states of S_{84} (a, b). The blue

and green isosurfaces represent the positive and negative TEDM, respectively, and the yellow and purple isosurfaces represent the positive and negative TMDM, respectively.

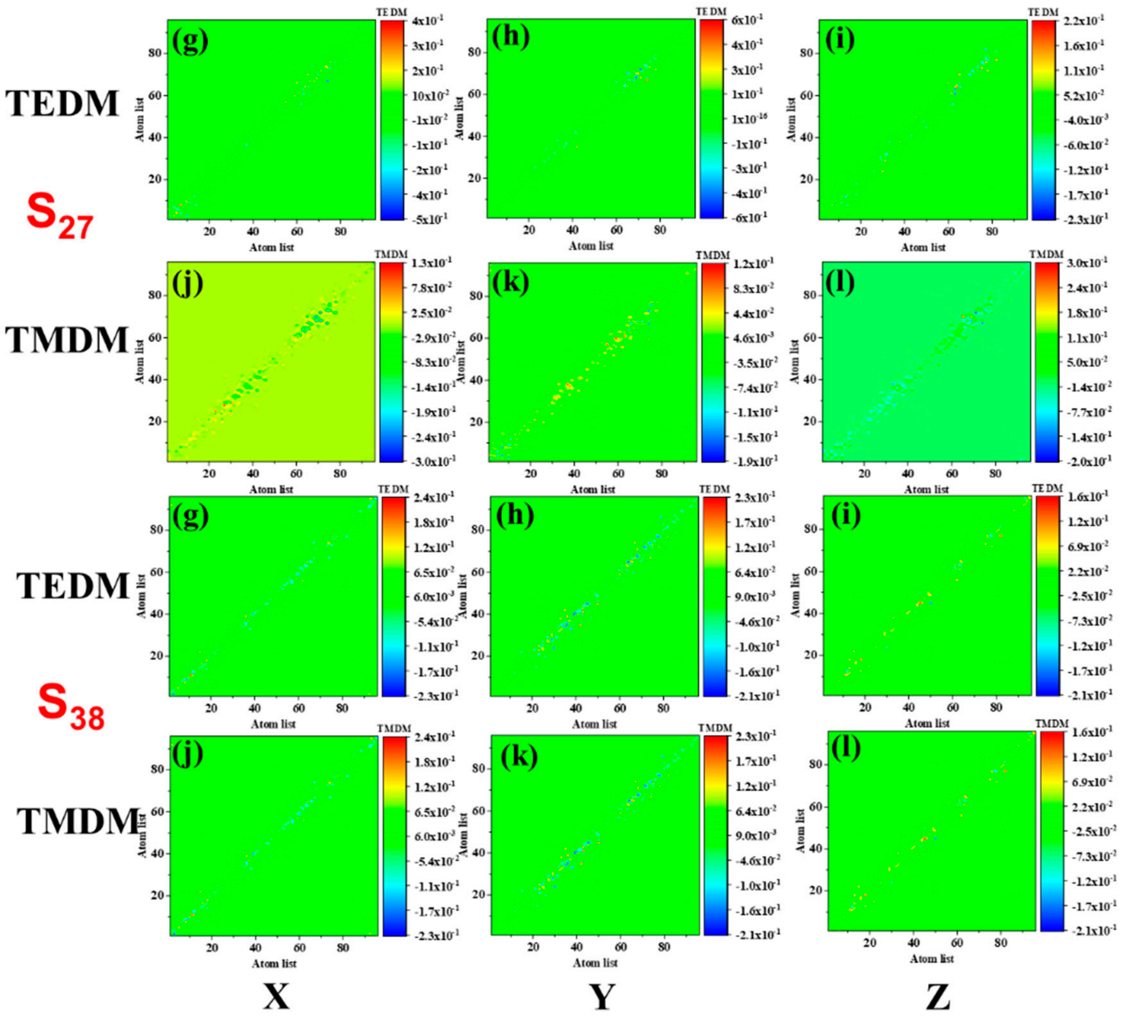

Figure S17. TDM diagrams of TEDM/TMDM of MCNB2 under S_{27} and S_{38}.

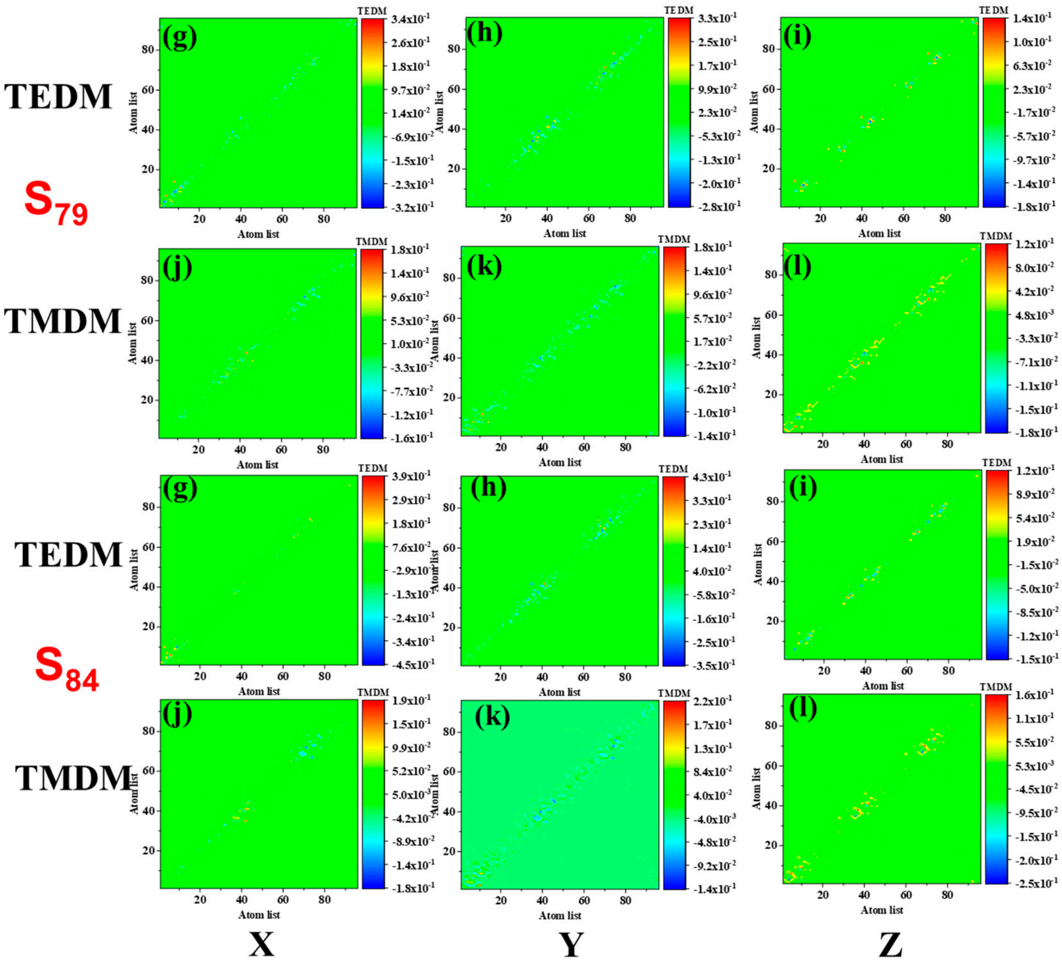

Figure S18. TDM diagrams of TEDM/TMDM of MCNB2 under S_{79} and S_{84}.
